# Development of BK polyomavirus-associated nephropathy risk prediction in kidney transplant recipients

**DOI:** 10.1080/0886022X.2025.2509785

**Published:** 2025-05-29

**Authors:** Junji Yamauchi, Katalin Fornadi, Divya Raghavan, Duha Jweehan, Suayp Oygen, Silviana Marineci, Michelle Buff, Michael Fenlon, Motaz Selim, Michael Zimmerman, Miklos Z. Molnar

**Affiliations:** ^a^ Division of Nephrology & Hypertension, Department of Internal Medicine, Spencer Fox Eccles School of Medicine at the University of Utah, Salt Lake City, UT, USA; ^b^ Division of Transplantation and Advanced Hepatobiliary Surgery, Department of Surgery, Spencer Fox Eccles School of Medicine at the University of Utah, Salt Lake City, UT, USA

**Keywords:** BK polyomavirus, BK polyomavirus-associated nephropathy, kidney transplantation, risk prediction, integer risk score

## Abstract

**Background:**

With the development of potential prevention therapies for BK polyomavirus (BKPyV)-associated nephropathy (BKPyVAN), risk prediction models are needed to identify kidney transplant recipients at high risk for BKPyVAN.

**Methods:**

This single-center retrospective study aimed to develop a risk prediction model and an integer-based risk score for BKPyVAN development, defined as plasma BKPyV-DNA >10,000 copies/mL and/or biopsy-proven BKPyVAN, within 1-year post-transplant, using donor and recipient characteristics at the time of transplantation. We randomly split patients into development and validation cohorts and applied logistic regression with backward selection to identify significant variables. Model performance was evaluated using the area under the receiver-operating characteristic curve (AUC) and calibration plots.

**Results:**

This study included 560 patients, of whom 75 (13%) patients had BKPyVAN. Age >50 years, male sex, and prior kidney transplant were selected for the final model. The total integer score ranged from 0 to 4 points, with 1 point assigned for age >50 years and male sex, and 2 points for prior kidney transplant. The AUC was 0.65 in both development and validation cohorts. Calibration plots showed an incremental increase in risk with higher total scores. The integer score indicated that patients with a total score of 2 or higher (i.e. males aged >50 years or those with prior kidney transplants) have a predicted risk of 20% or greater.

**Conclusion:**

Although the AUC was suboptimal, the results suggest that our model may still be valuable for identifying high-risk patients.

## Introduction

BK polyomavirus (BKPyV), the etiological agent of BKPyV-associated nephropathy (BKPyVAN), remains a significant threat to kidney transplant recipients. BKPyV replication most commonly occurs during the first year of post-transplant [1,2]. Plasma BKPyV-DNA, which is detected in ∼10–20% of recipients, almost invariably precedes the development of BKPyVAN [3–5]. BKPyVAN eventually develops in 1–10% of recipients and can lead to graft dysfunction and even graft failure [6–8]. Early diagnosis and intervention are critically important because BKPyVAN causes irreversible interstitial fibrosis and tubular atrophy [9,10]. Specific antiviral therapy for BKPyV-DNAemia or BKPyVAN has not been established, and thus immunosuppression reduction remains the mainstay of management [11–14]. While BKPyVAN is based on the histopathological diagnosis, plasma BKPyV-DNA of >10,000 copies/mL is widely adopted for clinical diagnosis as presumptive BKPyVAN [11,13,15]. Plasma BKPyV-DNA of >10,000 copies/mL has also been reported to correlate with a lower treatment response to immunosuppression reduction [7,16].

To date, risk prediction for BKPyVAN development has not been established, despite reports of various risk factors, including donor (donor type and urinary BKPyV shedding), recipient (older age, male sex, prior history of kidney transplantation, lymphocyte-depleting antibody induction, tacrolimus, ureteral stent placement, and ABO blood type incompatibility), and BKPyV factors (anti-BKPyV IgG level, genotypes, serotype, and genotype mismatch between the donor and recipient) [13,17–19]. Because BKPyVAN prevention therapies, such as intravenous immunoglobulin and anti-BKPyV monoclonal antibodies, are currently under development, establishing a BKPyVAN risk prediction model is essential to identify high-risk patients who may benefit most from these interventions [20,21]. Moreover, as BKPyV-DNAemia most commonly occurs within the first few months posttransplant, prediction models based on parameters at the time of or shortly after transplantation are crucial for the effective implementation of preventive therapies [2,3]. Therefore, we aimed to develop a risk prediction model for BKPyVAN development using clinical data available at the time of kidney transplantation.

## Materials and methods

### Data source and study population

**Figure 1.**
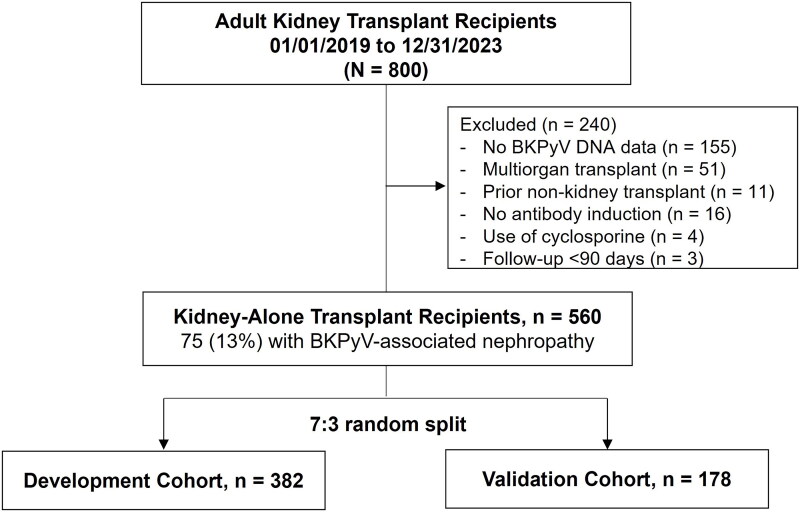
Study flowchart. BKPyV: BK polyomavirus.

This retrospective study included adult (≥18 years of age) patients who underwent kidney-alone transplantation at the University of Utah hospital from January 2019 to December 2023 (Figure 1). We only performed ABO-compatible transplants. We excluded patients who underwent multi-organ transplantation or those who had a prior history of non-kidney solid organ transplantation. We also excluded patients who did not receive induction or received steroid-only induction or those who were administered cyclosporine as initial maintenance immunosuppression because these treatments are not used at our center as of 2025. Patients with a follow-up of <3 months (i.e. graft failure, death, or loss of follow-up within 3 months posttransplant) were excluded. We extracted patient data from the electronic health records of our hospital. The University of Utah Institutional Review Board approved the present study and granted an exemption from informed consent (IRB_00162331).

### Outcome

We aimed to develop and validate a risk prediction model for the development of presumptive or biopsy-proven BKPyVAN within 1-year post-transplant using donor and recipient variables available at the time of kidney transplantation. We also aimed to create an integer-based risk score to facilitate clinical application.

### Definition of BKPyVAN

In this study, we used plasma BKPyV-DNA of >10,000 copies/mL (presumptive BKPyVAN) and biopsy-proven BKPyVAN for clinical diagnosis of BKPyVAN, because we only performed for-cause biopsy for limited patients [11,15]. BKPyVAN was defined as positive if a patient had at least one plasma BKPyV-DNA of >10,000 copies/mL or biopsy-proven BKPyVAN detected within the first year posttransplant. Those who had only plasma BKPyV-DNA results of ≤10,000 copies/mL were adjudicated negative for BKPyVAN. Negative or unquantifiable BKPyV-DNA was treated as 0 copy/mL. Regular plasma BKPyV-DNA screening based on our center protocol was monthly for the first 9 months, followed by every 3 months until 2 years post-transplant. If plasma BKPyV-DNA exceeded 1000 copies/mL, testing was repeated every 2 weeks. Biopsy-proven BKPyVAN was diagnosed according to the Banff 2019 classification [15].

### Potential predictors for BKPyVAN

We selected predictor candidates based on published literature and theoretical considerations [11,13]. We evaluated the following variables: recipient factors (age, sex, race, body-mass index, diabetes as cause of kidney failure, dialysis duration, prior kidney transplant, human leucocyte antigen [HLA] mismatch, lymphocyte-depleting antibody induction [anti-thymocyte globulin or alemtuzumab] *vs.* basiliximab, tacrolimus *vs.* belatacept for initial maintenance immunosuppression, ureteral stent placement, and delayed graft function defined as any dialysis in the first week post-transplant) and donor factors (donor type [deceased *vs.* living] and cold ischemia time). Continuous variables were categorized using the following cutoff values for logistic regression analysis: older age (age of 50 years), obesity (body mass index of 30 kg/m^2^), dialysis duration (preemptive and 3 years), and long cold ischemia time (24 h).

### Model development

We randomly split the dataset into a development cohort (70%) and a validation cohort (30%). Univariate and multivariate logistic regression analysis was performed for the development cohort to evaluate the associations between candidate predictors and clinical diagnosis of BKPyVAN. We applied multivariate logistic regression with backward selection with a *p*-value threshold of 0.1 to identify predictive variables from candidate predictors. The final logistic regression model was constructed using selected variables.

### Evaluation of model performance

The discrimination ability of the final logistic regression model was evaluated using the area under the receiver operating characteristic curve (AUC). AUC values >0.7 are generally considered indicative of good discrimination. Calibration was assessed using the calibration plots and the Brier score. In calibration plots, predicted probabilities were grouped into deciles and compared with observed event rates. The Brier score quantifies overall calibration by measuring the mean squared difference between predicted probabilities and observed outcomes. A lower Brier score indicates better calibration, with values higher than 0.25 suggesting poor calibration. We conducted these assessments on development and validation cohorts.

### Sensitivity analysis

As a sensitivity analysis of the final model, we conducted a 5-fold cross-validation for the AUC using the cvauroc command in STATA. The whole cohort was randomly split into 5-folds, with each fold serving as a test set while the remaining were used for training. The cross-validated AUC computed for each fold was averaged across the folds, and a bias-corrected 95% confidence interval (CI) was calculated *via* bootstrap procedure. Additionally, we developed a logistic regression model *via* backward selection with a cutoff of *p* < 0.2, instead of *p* < 0.1, and compared the AUC and the Brier score with those of the final model. As an additional sensitivity analysis, we evaluated the performance of the final model in patients with complete follow-up data to the end of the first-year post-transplant (complete 1-year follow-up cohort). We also developed the logistic regression model using the whole complete 1-year follow-up cohort and compared its performance with the final model.

### Creation and evaluation of the integer risk score

We created the integer-based risk score from the final logistic regression model. Each predictor’s coefficient (log-odds ratio) was divided by the smallest absolute coefficient in the model and rounded to the nearest integer. The total risk score for each patient was calculated as the sum of the points assigned for predictors. Discrimination ability was assessed by the AUC. Calibration was assessed by plotting the observed event rate against the total risk score.

### Other statistical analysis

Patient characteristics were summarized as mean ± standard deviation (*SD*) or median and interquartile range (IQR) for continuous variables and number (percent) for categorical variables. We compared continuous variables *via* the *t*-test or the Mann-Whitney *U* test and categorical variables *via* the Chi-square test, as appropriate. The two-sided *p*-value of <0.05 was considered statistically significant. All statistical analyses were conducted using STATA Version 18 (STATA Corporation, College Station, TX, USA).

## Results

### Patient characteristics

After exclusion, a total of 560 kidney transplant recipients were included in this study, of whom 75 (13%) patients had presumptive and/or biopsy-proven BKPyVAN within 1-year post-transplant (Figure 1). Table 1 shows patient characteristics. Recipients in the BKPyVAN-positive group tended to be older than those in the BKPyVAN-negative group (mean ± *SD*, 54 ± 15 *vs.* 50 ± 15 years, *p* = 0.066) and more patients were aged >50 years (68 *vs.* 54%, *p* = 0.021). Male sex was more frequent in the BKPyVAN-positive group (75 *vs.* 56%, *p* = 0.002). Recipient race, body mass index, dialysis duration, cause of kidney failure, and HLA mismatch were not significantly different between groups. The prior history of kidney transplants was more frequent in the BKPyVAN-positive group (16 *vs.* 7%, *p* = 0.006). Of 45 recipients with prior kidney transplants, four patients had experienced BKPyVAN in prior transplants (two patients in each group). The type of induction agent was similar between groups (lymphocyte-depleting antibody, 92 *vs.* 90%; basiliximab, 8 *vs.* 10%; *p* = 0.60). Belatacept/tacrolimus use as initial maintenance immunosuppression was also not statistically different (tacrolimus, 85 *vs.* 80%; belatacept, 15 *vs.* 20%; *p* = 0.24). All recipients received mycophenolate and prednisone. Ureteral stent was less frequently placed in the BKPyVAN-positive group (72 *vs.* 82%, *p* = 0.040), which was inconsistent with previous reports [3,22]. Delayed graft function was observed in 31 (6%) and the frequency was similar in BKPyVAN-positive and negative groups (2/75 [3%] *vs.* 29/485 [6%], *p* = 0.24). Regarding the donor characteristics, no significant difference was observed in donor type (deceased donor, 75 *vs.* 67%, *p* = 0.17) and cold ischemia time (mean ± *SD*, 12 ± 9 *vs.* 13 ± 8 h, *p* = 0.62) between groups.

**Table 1.** Patient characteristics of the whole cohort.

Characteristic	Total	BKPyVAN negative	BKPyVAN positive	*p*-Value
	*N* = 560	*N* = 485	*N* = 75	
Recipient				
Recipient age (years)	51 (15)	50 (15)	54 (15)	0.066
Age (4 categories)				0.14
≤40 years	166 (30%)	150 (31%)	16 (21%)	
40<–50 years	82 (15%)	74 (15%)	8 (11%)	
50<–60 years	129 (23%)	109 (22%)	20 (27%)	
>60 years	183 (33%)	152 (31%)	31 (41%)	
Age (2 categories)				0.021
≤50 years	248 (44%)	224 (46%)	24 (32%)	
>50 years	312 (56%)	261 (54%)	51 (68%)	
Recipient sex				0.002
Female	233 (42%)	214 (44%)	19 (25%)	
Male	327 (58%)	271 (56%)	56 (75%)	
Race				0.54
White	363 (65%)	314 (65%)	49 (65%)	
Black	9 (2%)	6 (1%)	3 (4%)	
Hispanic	80 (14%)	71 (15%)	9 (12%)	
Asian	23 (4%)	19 (4%)	4 (5%)	
American Indian/Alaska Native	19 (3%)	15 (3%)	4 (5%)	
Native Hawaiian/Pacific Islander	22 (4%)	20 (4%)	2 (3%)	
Other	17 (3%)	16 (3%)	1 (1%)	
Unknown	27 (5%)	24 (5%)	3 (4%)	
Race category				0.92
White	363 (65%)	314 (65%)	49 (65%)	
Other	197 (35%)	171 (35%)	26 (35%)	
Body mass index (kg/m^2^)	28.5 (5.3)	28.5 (5.4)	28.4 (5.1)	0.92
Body mass index (4 categories)				0.95
<25 kg/m^2^	152 (27%)	133 (27%)	19 (25%)	
25–<30 kg/m^2^	194 (35%)	166 (34%)	28 (37%)	
30–<35 kg/m^2^	133 (24%)	115 (24%)	18 (24%)	
≥35 kg/m^2^	81 (14%)	71 (15%)	10 (13%)	
Body mass index (2 categories)				0.87
<30 kg/m^2^	346 (62%)	299 (62%)	47 (63%)	
≥30 kg/m^2^	214 (38%)	186 (38%)	28 (37%)	
Dialysis duration (years)	3 (1–5)	3 (1–5)	4 (2–5)	0.23
Dialysis duration category (3 categories)				0.26
Preemptive	114 (20%)	101 (21%)	13 (17%)	
<3 years	240 (43%)	212 (44%)	28 (37%)	
≥3 years	206 (37%)	172 (35%)	34 (45%)	
Cause of kidney failure				0.28
Diabetes	159 (28%)	139 (29%)	20 (27%)	
Hypertension	80 (14%)	64 (13%)	16 (21%)	
Glomerulonephritis	119 (21%)	102 (21%)	17 (23%)	
Cystic disease	54 (10%)	50 (10%)	4 (5%)	
Others	148 (26%)	130 (27%)	18 (24%)	
Cause of kidney failure category				0.72
Other	401 (72%)	346 (71%)	55 (73%)	
Diabetes	159 (28%)	139 (29%)	20 (27%)	
HLA mismatch	4 (3–5)	4 (3–5)	4 (3–5)	0.82
HLA mismatch				0.60
0	24 (4%)	20 (4%)	4 (5%)	
1	13 (2%)	10 (2%)	3 (4%)	
2	30 (5%)	27 (6%)	3 (4%)	
3	104 (19%)	95 (20%)	9 (12%)	
4	126 (23%)	106 (22%)	20 (27%)	
5	187 (33%)	160 (33%)	27 (36%)	
6	76 (14%)	67 (14%)	9 (12%)	
HLA mismatch category				0.29
0–3 mismatch	171 (31%)	152 (31%)	19 (25%)	
4–6 mismatch	389 (69%)	333 (69%)	56 (75%)	
Prior kidney transplant	45 (8%)	33 (7%)	12 (16%)	0.006
BKPyVAN history in prior kidney transplant	4 (9%)	2 (6%)	2 (17%)	0.27
Induction antibody				0.60
Basiliximab	54 (10%)	48 (10%)	6 (8%)	
Anti-thymocyte globulin/alemtuzumab	506 (90%)	437 (90%)	69 (92%)	
Belatacept/tacrolimus				0.24
Belatacept	110 (20%)	99 (20%)	11 (15%)	
Tacrolimus	450 (80%)	386 (80%)	64 (85%)	
Ureteral stent placement	452 (81%)	398 (82%)	54 (72%)	0.040
Delayed graft function	31 (6%)	29 (6%)	2 (3%)	0.24
Donor				
Donor type				0.17
Living	180 (32%)	161 (33%)	19 (25%)	
Deceased	380 (68%)	324 (67%)	56 (75%)	
Cold ischemia time (h)	12 (9)	12 (9)	13 (8)	0.62
Cold ischemia time (4 categories)				0.56
<12 h	280 (50%)	242 (50%)	38 (51%)	
12–<18 h	123 (22%)	106 (22%)	17 (23%)	
18–<24 h	101 (18%)	91 (19%)	10 (13%)	
≥24 h	56 (10%)	46 (9%)	10 (13%)	
Cold ischemia time (2 categories)				0.30
<24 h	504 (90%)	439 (91%)	65 (87%)	
≥24 h	56 (10%)	46 (9%)	10 (13%)	
BK polyomavirus				
Highest plasma BKPyV DNA within 1 year (copies/mL)	0 (0–480)	0 (0–0)	190,000 (51,400–833,000)	<0.001
Plasma BKPyV DNA category (highest within 1 year)				<0.001
Not detected or unquantifiable	388 (69%)	388 (80%)	0 (0%)	
≤1000 copies/mL	46 (8%)	46 (9%)	0 (0%)	
1000<–10,000 copies/mL	51 (9%)	51 (11%)	0 (0%)	
>10,000 copies/mL	75 (13%)	0 (0%)	75 (100%)	
Days from transplant to first plasma BKPyV DNA >10,000 copies/mL	104 (77–167)	–	104 (77–167)	–
Kidney biopsy performed within 1 year	211 (38%)	170 (35%)	41 (55%)	<0.001
Kidney biopsy performed during BKPyV DNA >10,000 copies/mL	33 (44%)	–	33 (44%)	–
Biopsy-proven BKPyVAN	27 (5%)	0 (0%)	27 (36%)	–

BKPyV: BK polyomavirus; BKPyVAN: BK polyomavirus-associated nephropathy.

Values are expressed as mean (standard deviation), median (interquartile range), or number (%). Continuous variables were compared *via t*-tests or Mann-Whitney *U* tests. Categorical variables were compared *via* Chi-square tests.

The median highest BKPyV-DNA within 1-year posttransplant was 190,000 (IQR, 51,400–833,000) and 0 (0–0) copies/mL in the BKPyVAN-positive and negative groups, respectively. In the BKPyVAN-positive group, all patients had BKPyV-DNA >10,000 copies/mL, and the median time from transplant to the first detection of BKPyV-DNA >10,000 copies/mL was 104 (IQR, 77–167) days. Of 33 (44%) patients who underwent kidney biopsy when BKPyV-DNA exceeded 10,000 copies/mL, 27 patients were diagnosed with biopsy-proven BKPyVAN. In the BKPyVAN-negative group, no biopsy-proven BKPyVAN was observed among 170 (35%) patients who experienced kidney biopsy in the first year.

### Development of the BKPyVAN risk prediction model

The whole cohort was randomly split into development and validation cohorts (patient characteristics are available in Tables S1 and S2). We performed univariate and multivariate logistic regression analyses for the development cohort (Table 2). We did not include ureteral stent placement (due to inconsistency with previous reports) and delayed graft function (due to the small event number) in multivariate analysis. Age of >50 years, male sex, and prior kidney transplant had a *p*-value <0.1 in both analyses. These three variables remained in the final model after backward selection: age of >50 years (odds ratio: 1.89 [95% CI, 1.00–3.57], *p* = 0.050), male sex (2.13 [95% CI, 1.10–4.10], *p* = 0.025), and prior kidney transplant (2.94 [95% CI, 1.25–6.95], *p* = 0.014). The predicted probability based on the final model was:

Predicted probability = 1/{1– exp [2.832–0.635(age >50 years)–0.754(male sex)– 1.080(prior kidney transplant)]}

**Table 2.** Logistic regression analysis for BK polyomavirus-associated nephropathy development within 1 year posttransplant.

Covariates	Univariate			Multivariate			Final model (*p* < 0.1)		
	Odds ratio	95% CI	*p*-Value	Odds ratio	95% CI	*p*-Value	Odds ratio	95% CI	*p*-Value
Age >50 years (*vs.* ≤50 years)	1.82	(0.98, 3.37)	**0.057**	2.06	(1.04, 4.09)	**0.039**	1.89	(1.00, 3.57)	**0.050**
Male (*vs.* female)	2.20	(1.15, 4.20)	**0.017**	2.28	(1.16, 4.49)	**0.017**	2.13	(1.10, 4.10)	**0.025**
Race other (*vs.* White)	1.04	(0.57, 1.91)	0.899	1.14	(0.57, 2.26)	0.707			
Body mass index ≥30 kg/m^2^ (*vs.* <30 kg/m^2^)	1.10	(0.61, 1.98)	0.751	1.10	(0.59, 2.05)	0.761			
Kidney failure from diabetes (*vs.* other)	1.10	(0.58, 2.09)	0.777	0.93	(0.45, 1.92)	0.853			
Dialysis duration (*vs.* preemptive)									
<3 years	0.92	(0.40, 2.12)	0.836	0.73	(0.29, 1.86)	0.507			
≥3 years	1.49	(0.65, 3.40)	0.347	1.11	(0.41, 3.01)	0.832			
HLA mismatch (*vs.* 0–3 mismatch)									
4–6 mismatch	1.07	(0.57, 2.01)	0.836	1.02	(0.51, 2.03)	0.961			
Prior kidney transplant, yes (*vs.* no)	2.49	(1.09, 5.67)	**0.030**	2.85	(1.13, 7.19)	**0.027**	2.94	(1.25, 6.95)	**0.014**
Deceased donor (*vs.* living donor)	1.46	(0.76, 2.80)	0.257	1.55	(0.67, 3.57)	0.301			
Cold ischemia time ≥24 h (*vs.* <24 h)	1.28	(0.54, 3.05)	0.580	1.24	(0.49, 3.15)	0.655			
Anti-thymocyte globulin/alemtuzumab (*vs.* basiliximab)	1.27	(0.43, 3.77)	0.661	1.34	(0.42, 4.25)	0.623			
Tacrolimus (*vs.* belatacept)	1.41	(0.63, 3.14)	0.398	1.66	(0.71, 3.91)	0.243			
Ureteral stent, yes (*vs.* no)	0.59	(0.30, 1.14)	0.114	–	–	–			
Delayed graft function, yes (*vs.* no)	0.30	(0.04, 2.26)	0.241	–	–	–			

Univariate and multivariate logistic regression was performed for the development cohort. Backward selection using a *p*-value threshold of 0.1 was applied to identify predictive variables from candidate predictors. The final logistic regression model was constructed using selected variables. Bold values denote *p*-values <0.1.

### Evaluation of the final logistic regression model

**Figure 2.**
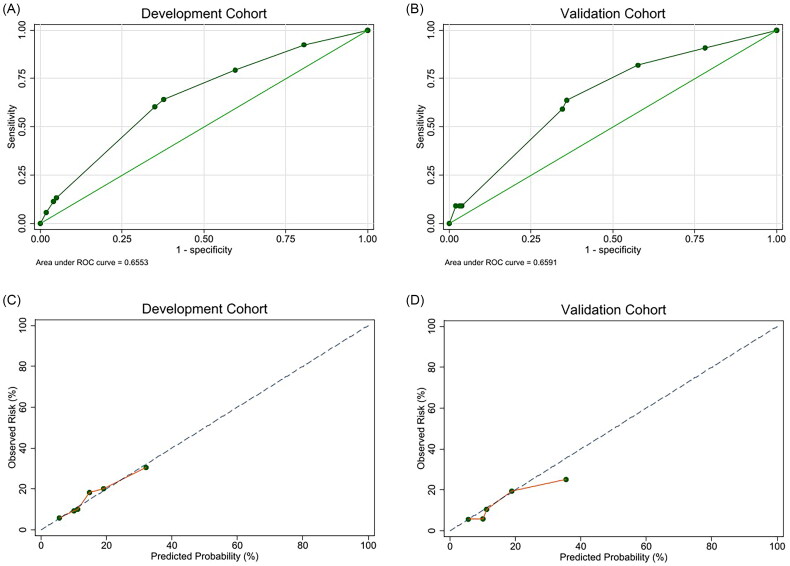
Performance of the final logistic regression model. (A,B) Receiver-operating characteristic curves to evaluate the discrimination ability of the final model for BK polyomavirus-associated nephropathy development in (A) development (AUC = 0.66 [95% CI, 0.58–0.73]) and (B) validation cohorts (AUC = 0.66 [95% CI, 0.54–0.77]). (C,D) Calibration plots to assess the agreement between predicted and observed probabilities in (C) development and (D) validation cohorts. The dashed line indicates the perfect agreement between predicted and observed probabilities. The solid line represents the model’s actual performance. A closer alignment of the dashed and solid lines suggests better calibration.

The AUC for the discrimination assessment was 0.66 (95% CI, 0.58–0.73) in the development cohort and 0.66 (95% CI, 0.54–0.77) in the validation cohort (Figure 2). Calibration plots demonstrated good calibration for both development and validation cohorts, with good Brier scores (0.115 [95% CI, 0.091–0.139] and 0.104 [95% CI, 0.070–0.138]).

### Sensitivity analysis

**Figure 3.**
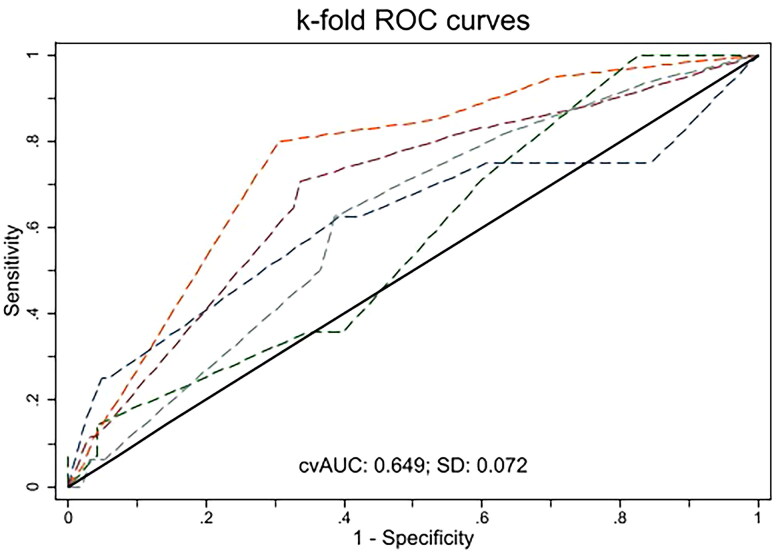
Cross-validation receiver-operating characteristic curve of the final logistic regression model. The area under the receiver-operating characteristic curve (AUC) was evaluated *via* 5-fold cross validation. The whole cohort (*n* = 560) was randomly split into 5-folds, with each fold serving as a test set while the remaining were used for training. The cross-validated AUC (cvAUC) computed for each fold was averaged across folds. cvAUC = 0.65 (95% CI, 0.53–0.68).

The 5-fold cross-validation analysis for the final model demonstrated an AUC comparable to the development and validation cohorts (0.65 [95% CI, 0.53–0.68]; Figure 3). When backward selection with a cutoff *p*-value <0.2 was applied, donor type was additionally selected (odds ratio for deceased *vs.* living, 1.60 [95% CI, 0.82–3.12], *p* = 0.17; Table S3). Model performance did not improve, however, with an AUC of 0.67 (*p* = 0.15 *vs.* final model) in the development cohort and 0.66 (*p* = 0.92) in the validation cohort (Figure S1). The Brier score (0.114 [95% CI, 0.091–0.138] in the development cohort and 0.103 [95% CI, 0.070–0.137] in the validation cohort) and the cross-validated AUC (0.65 [95%CI, 0.55–0.70]; Figure S2) were also nearly identical to those of the final model.

The complete 1-year follow-up cohort included 488 patients. The performance of the final model in this cohort was consistent with the primary analysis (AUC, 0.66 [95% CI, 0.59–0.73]; Brier score, 0.115 [95% CI, 0.094–0.136]; Figure S3). The logistic regression model developed using this cohort selected tacrolimus use as an additional predictor alongside those in the final model (odds ratio for tacrolimus *vs.* belatacept, 1.96 [95% CI, 0.89–4.33], *p* = 0.097; Table S4). However, its performance did not differ from the final model, supporting the validity of the final model (Figures S4–S6).

**Figure 4.**
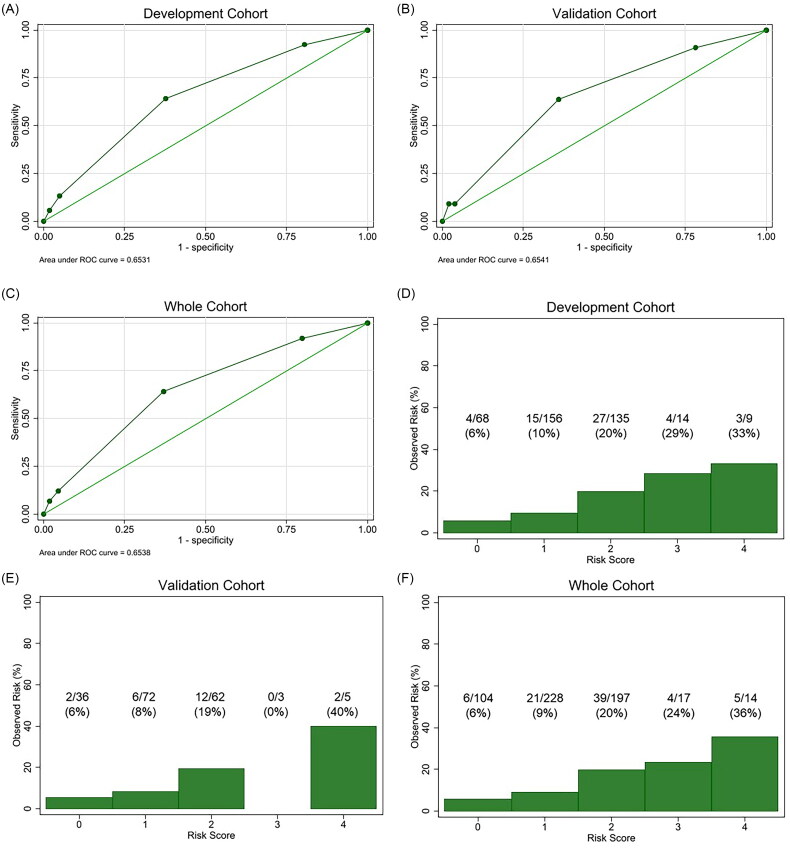
Performance of the integer risk score. (A–C) Receiver-operating characteristic curves to evaluate the discrimination ability of the integer risk score for BK polyomavirus-associated nephropathy (BKPyVAN) development for (A) development (AUC = 0.65 [95% CI, 0.58–0.73]), (B) validation (AUC = 0.65 [95% CI, 0.54–0.77]), and (C) whole cohorts (AUC = 0.65 [95% CI, 0.59–0.72]). (D–F) Calibration assessed by plotting the observed event rates against risk score categories for (D) development, (E) validation, and (F) whole cohorts. The numbers at the bottom represent the number and percentage of patients with BKPyVAN in each score category.

### Development and validation of the integer risk score

Finally, we created an integer risk score from the final logistic regression model. The total score ranged from 0 to 4 points, with 1 point assigned for age >50 years and male sex, and 2 points for prior kidney transplant (Table 3). The AUC was 0.65 (95% CI, 0.58–0.73), 0.65 (95% CI, 0.54–0.77), and 0.65 (95% CI, 0.59–0.72) in the development, validation, and whole cohorts, respectively (Figures 4(A–C)). The calibration plots showed an incremental increase in risk with higher total scores, although the total score of 3 in the validation cohort had no events, due to the small sample size (Figures 4(D–F)). The sensitivity, specificity, and positive likelihood ratio were 92.0%, 20.2%, and 1.15 with a cutoff point of ≥1; 64.0%, 62.9%, and 1.72 with a cutoff point of ≥2; 12.0%, 95.5%, and 2.65 with a cutoff point of ≥3; and 6.7%, 98.1%, and 3.59 with a cutoff point of ≥4, respectively (Table 4). Based on the prevalence of BKPyVAN in the whole cohort (13%) and the positive likelihood ratios, the integer score indicated that patients with a total score of ≥1 (i.e. age >50 years, male sex, or prior kidney transplant), ≥2 (i.e. males aged >50 years or those with prior kidney transplant), ≥3 (i.e. prior kidney transplant plus age >50 years and/or male sex), and ≥4 (i.e. males aged >50 years with prior kidney transplant) have a predicted risk of 15, 20, 28, and 35%, respectively.

**Table 3.** Integer risk score.

Predictor	Coefficient (log-odds ratio)	Integer score
Age >50 years	0.635	1
Male	0.754	1
Prior kidney transplant	1.080	2
Total score	–	0–4

The integer-based risk score derived from the coefficients (log-odds ratios) of the final logistic regression model. Each predictor’s coefficient was divided by the smallest absolute coefficient and rounded to the nearest integer. The total score ranges from 0 to 4 points.

**Table 4.** Performance of the integer risk score for the whole cohort.

Cut point	Sensitivity	Specificity	LR+	LR−	Post-test probability
≥1	92.0%	20.2%	1.15	0.40	15%
≥2	64.0%	62.9%	1.72	0.57	20%
≥3	12.0%	95.5%	2.65	0.92	28%
≥4	6.7%	98.1%	3.59	0.95	35%

LR+: positive likelihood ratio; LR−: negative likelihood ratio.

The post-test probability was calculated using the overall prevalence of BK polyomavirus-associated nephropathy (13%) and LR+.

## Discussion

Predicting the development of BKPyVAN after kidney transplantation remains a challenge. In this study, we developed a risk prediction model for BKPyVAN development within the first year posttransplant based on the variables available at the time of transplant, with the goal of identifying high-risk patients who may benefit from prevention therapies. Age >50 years, male sex, and prior kidney transplant were selected for the final model as significant predictors. The integer risk score indicated that a total score of ≥2 (males aged >50 years and those with prior kidney transplants) have a predicted risk of 20% or greater. Although the discrimination of the final model was suboptimal, its calibration was acceptable.

Numerous risk factors for BKPyVAN have been documented, however, those reaching statistical significance vary across studies, with only a few consistently identified in each publication [13]. Nonetheless, our cohort largely reflected the characteristics of patients with BKPyVAN described in previous reports, including older age, male sex, and prior kidney transplant. Other reported risk factors, such as deceased donor status and longer dialysis vintage, also exhibited similar trends but did not reach statistical significance. In our study, ureteral stent placement was associated with a lower risk, contradicting previous studies that reported a higher risk in patients with a ureteral stent [3,22]. Short-term stent placement (<3 weeks) was reported to not significantly increase BKPyVAN risk, whereas ureteral stent retained for more than 3 weeks was associated with a higher risk [23]. Stents are typically removed within 3–4 weeks post-transplant at our center. Although the reasons for this finding are unclear, the results suggest that our stent management does not contribute to the increased risk of BKPyVAN.

Our final model identified older age, male sex, and prior kidney transplant as significant risk factors. In the sensitivity analysis, the use of deceased donor organ and tacrolimus (*vs.* belatacept) were additionally selected as predictors; however, their *p*-values were marginal, and model performance remained unchanged, supporting the robustness of the final model. Moreover, although tacrolimus has been associated with a higher risk of BKPyVAN compared to cyclosporine in previous studies, no significant difference has been observed between tacrolimus and belatacept [24–26]. The final model showed modest discrimination (AUC = 0.66). However, calibration was acceptable, with good agreement between predicted and observed risks. The Brier score of 0.10–0.12 also indicated reasonable overall accuracy. While the limited AUC suggests restricted discriminative power, the acceptable calibration supports its potential use for risk stratification.

We also created the integer risk score based on the final model. While the predicted BKPyVAN risk of patients with a total score of ≥1 (15%) was similar to the pretest probability in our cohort (13%), the posttest probability incrementally increased to 20, 28, and 35% for those with a total score of ≥2, ≥3, and ≥4, respectively, suggesting that our model would be useful to identify candidates for BKPyVAN prevention trials, which require the enrollment of high-risk patients to achieve appropriate statistical power. That said, the model was based on a limited set of predictors and showed suboptimal performance, underscoring the need for further refinement. Our results may suggest that relying solely on variables at the time of transplant is insufficient and that different modeling approaches and additional clinical variables are needed to enhance model performance. Fang et al. reported that dynamic risk prediction outperformed static prediction models in predicting BKPyV reactivation after kidney transplantation (AUC, 0.70 *vs.* 0.63) [27]. Their dynamic model estimated the risk within a specific posttransplant period by incorporating both static variables (i.e. pretransplant and early posttransplant data) and posttransplant parameters, such as graft function, urine protein, and tacrolimus concentrations. This approach is reasonable, given that posttransplant factors, such as graft rejection and immunosuppression intensity, are significant contributors to BKPyVAN development [11]. Although the dynamic model does not align with our study’s objective, it may be valuable depending on the trial design and usage of preventive therapies. Additionally, pretransplant anti-BKPyV antibody testing may enhance predictive performance because studies suggest that kidney recipients with low anti-BKPyV antibody titers have higher BKPyVAN risk and that intravenous immune globulin administration in these patients can increase antibody levels and reduce BKPyVAN risk [21,28–30]. However, further research is needed to incorporate antibody testing into clinical practice, as it is neither widely available nor recommended by current guidelines [11,13]. Donor BKPyV viruria has also been reported as a risk factor and may help predict BKPyVAN risk [31–33].

This study has important limitations. It was a single-center retrospective study with a predominantly white patient population. Most patients received lymphocyte-depleting antibody induction and tacrolimus-based maintenance immunosuppression. Therefore, generalizability may be limited. We did not perform external validation. Furthermore, the number of events were relatively small, and not all potential predictors were included in the analysis, which might have caused bias and contributed to suboptimal model performance. Thus, larger studies involving more diverse patient populations are needed to minimize bias and improve generalizability. External validation studies are also essential to confirm the model performance.

In conclusion, we developed a risk prediction model for BKPyVAN within 1 year posttransplant, identifying older age, male sex, and prior kidney transplant as significant contributors. While the model’s performance was suboptimal, the findings suggest that it may still be useful for identifying high-risk patients.

## Supplementary Material

Figures_TIF_R1.zip

BKVAN_Risk_Supplement_Tables_R1.docx
