# Supplementary figures and images for: Development of BK polyomavirus-associated nephropathy risk prediction in kidney transplant recipients
Source: Ren Fail. 2025 May 29;47(1):2509785. doi: 10.1080/0886022X.2025.2509785 (PMC12123896; doi:10.1080/0886022X.2025.2509785)

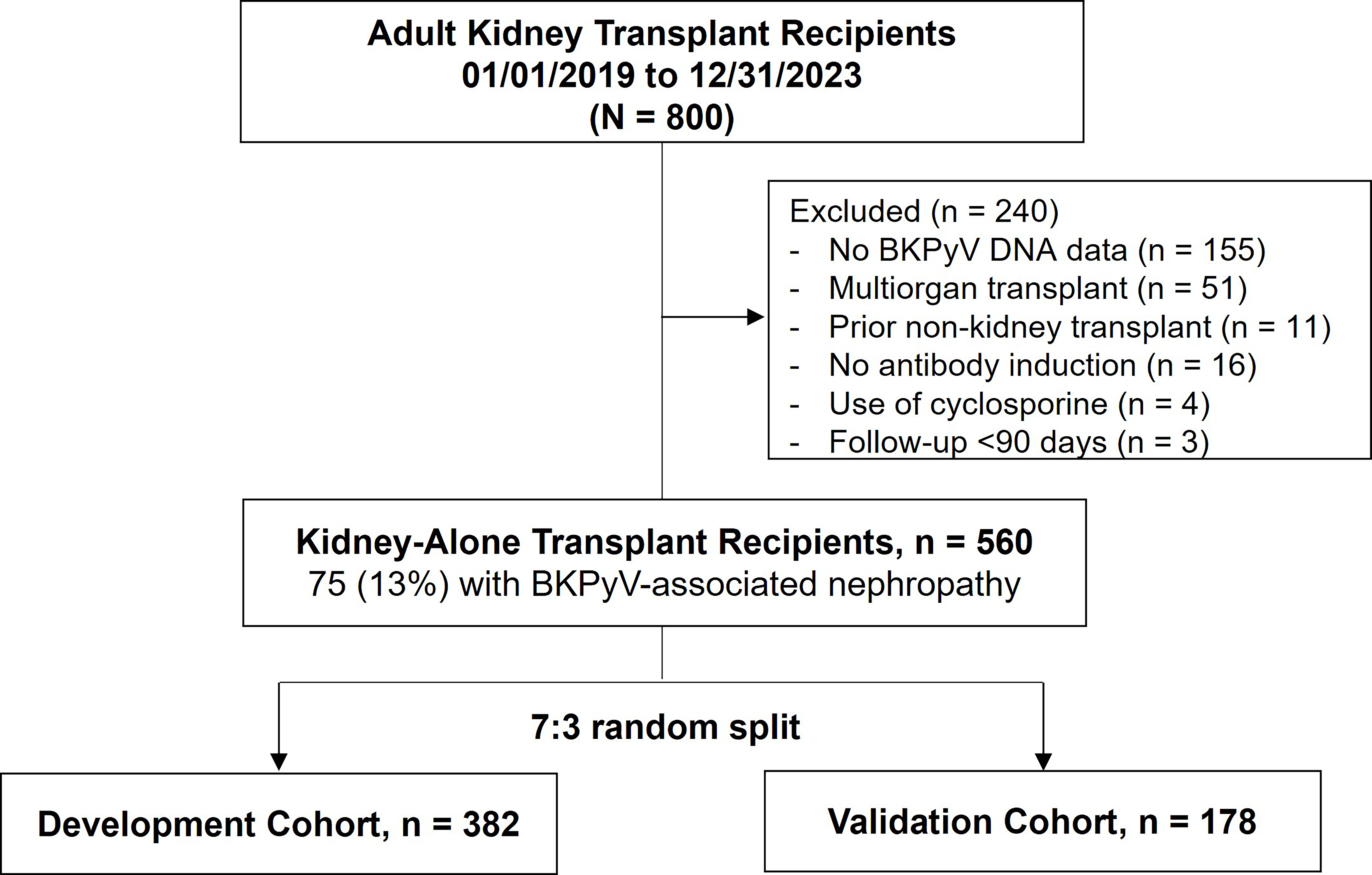

Supplement: Figures_TIF_R1.zip [file IRNF_A_2509785_SM5944.zip › Figures_TIF_R1/Figure1.tif]

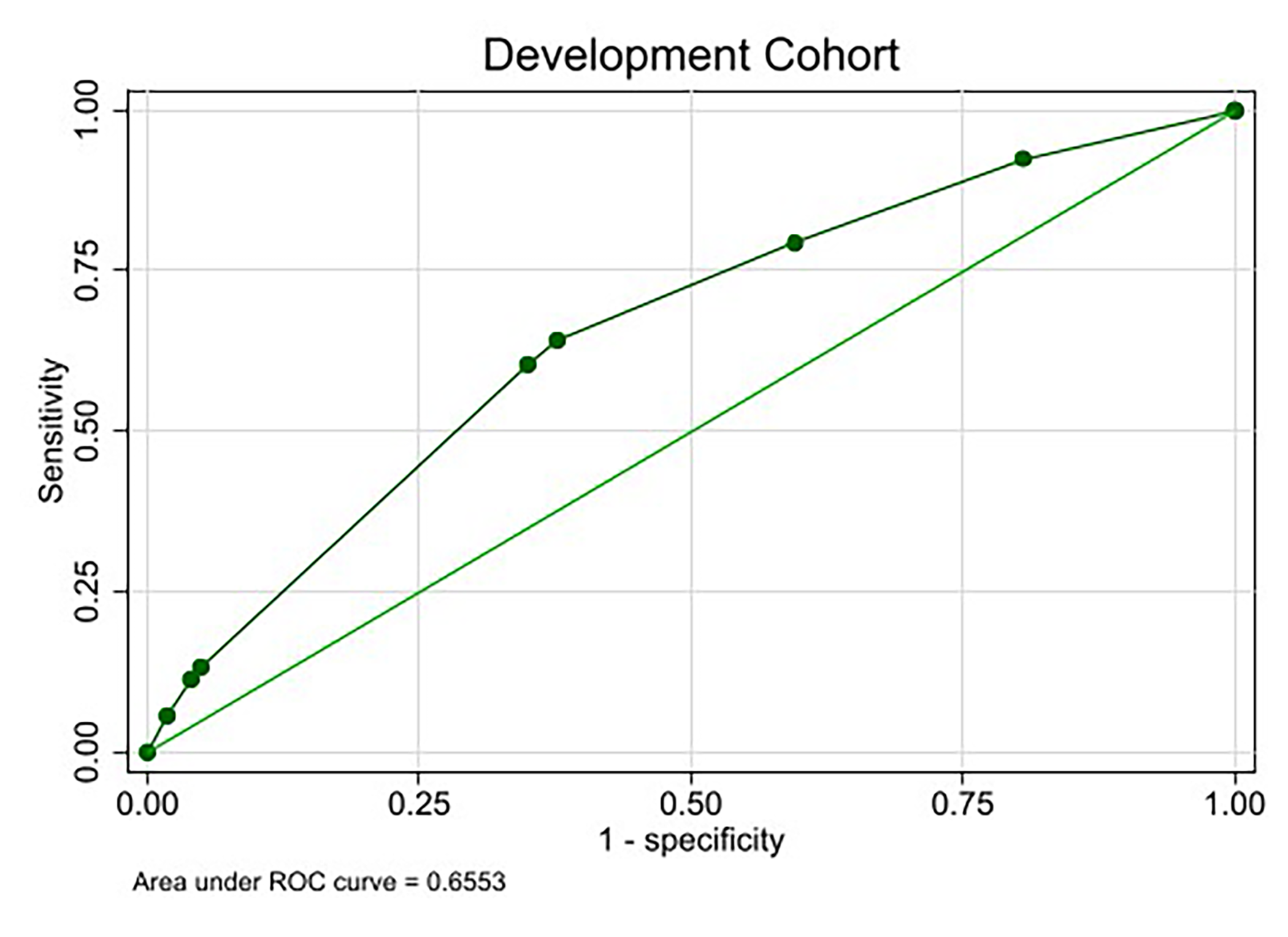

Supplement: Figures_TIF_R1.zip [file IRNF_A_2509785_SM5944.zip › Figures_TIF_R1/Figure2A.tif]

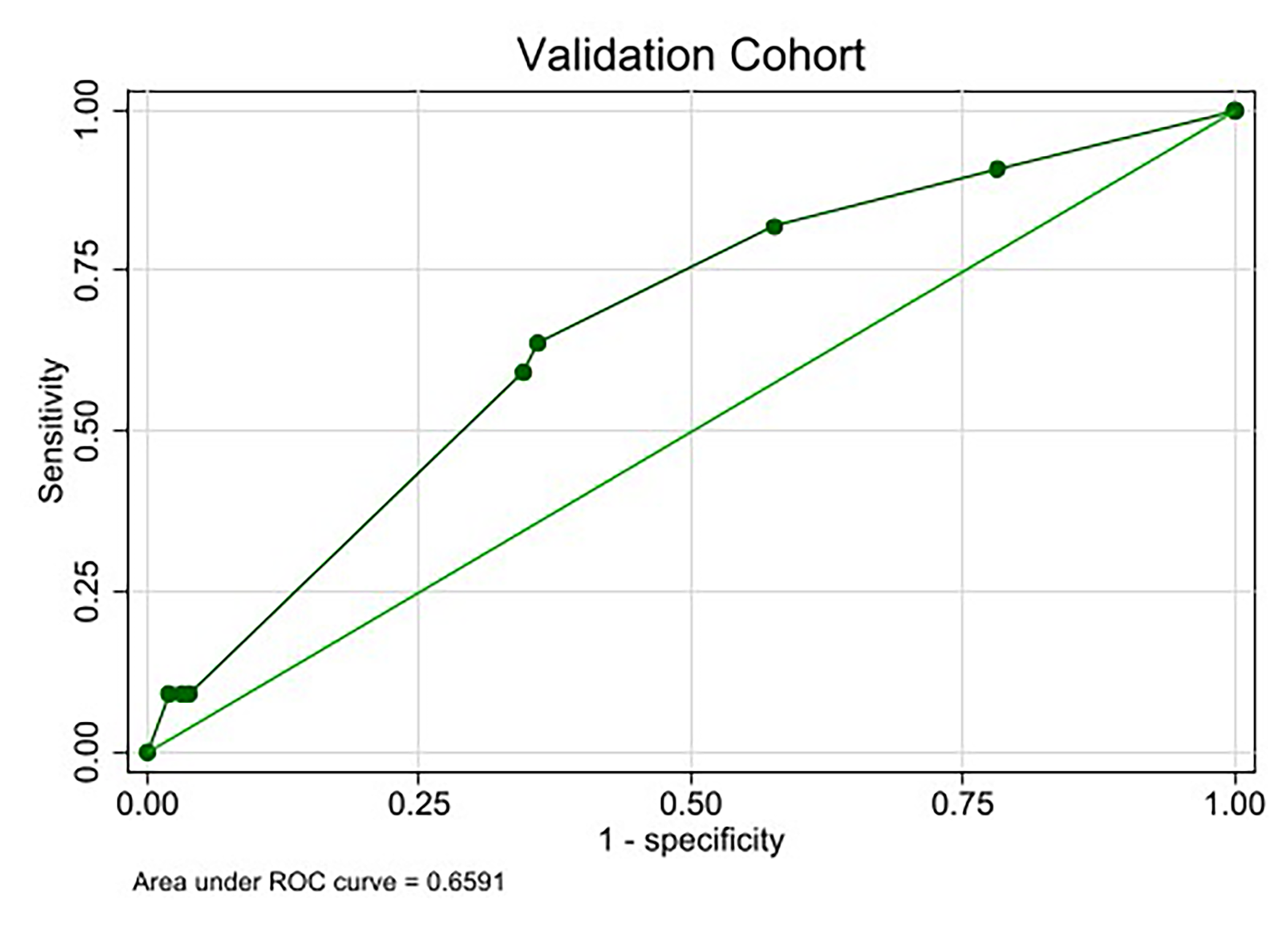

Supplement: Figures_TIF_R1.zip [file IRNF_A_2509785_SM5944.zip › Figures_TIF_R1/Figure2B.tif]

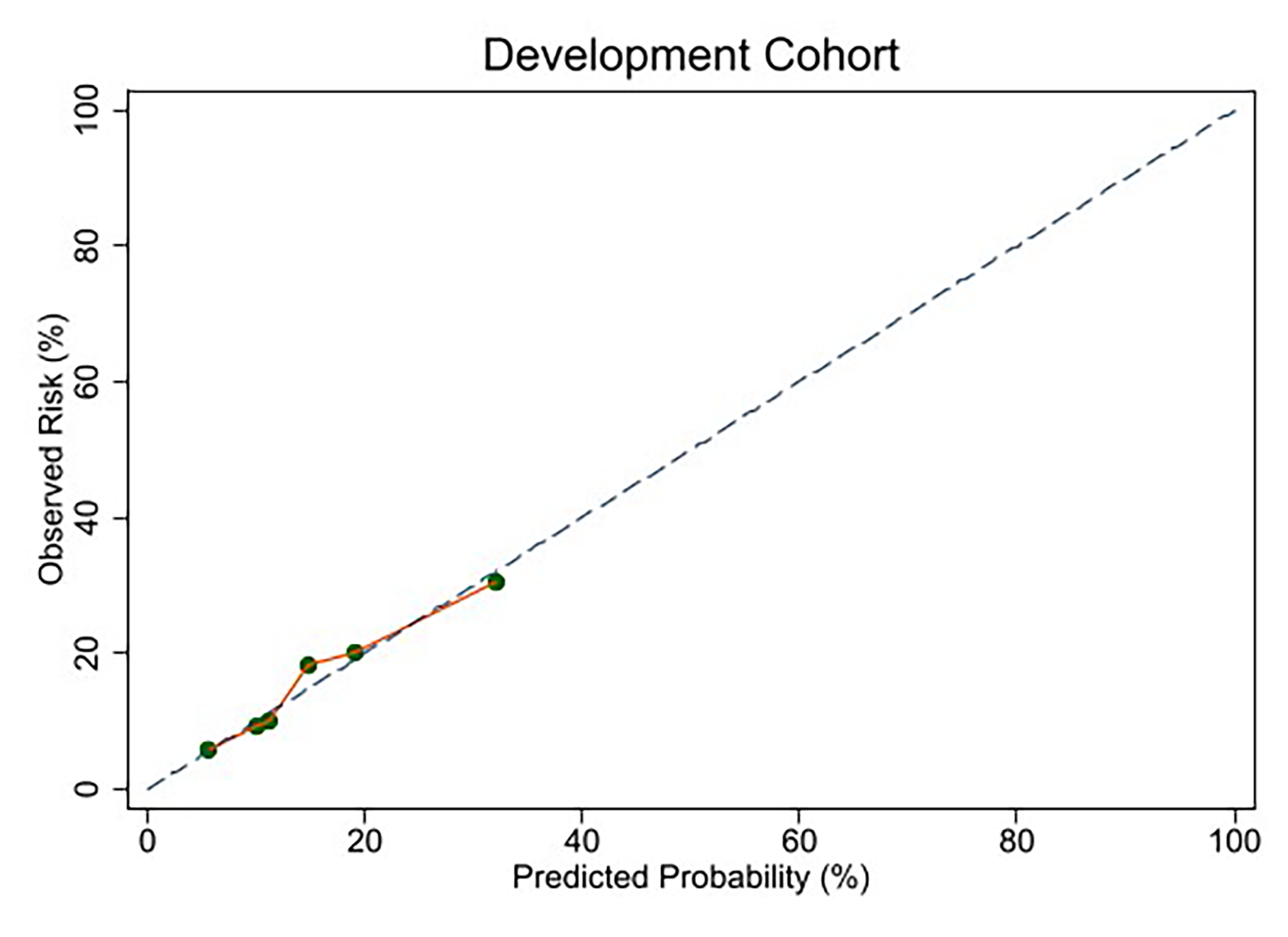

Supplement: Figures_TIF_R1.zip [file IRNF_A_2509785_SM5944.zip › Figures_TIF_R1/Figure2C.tif]

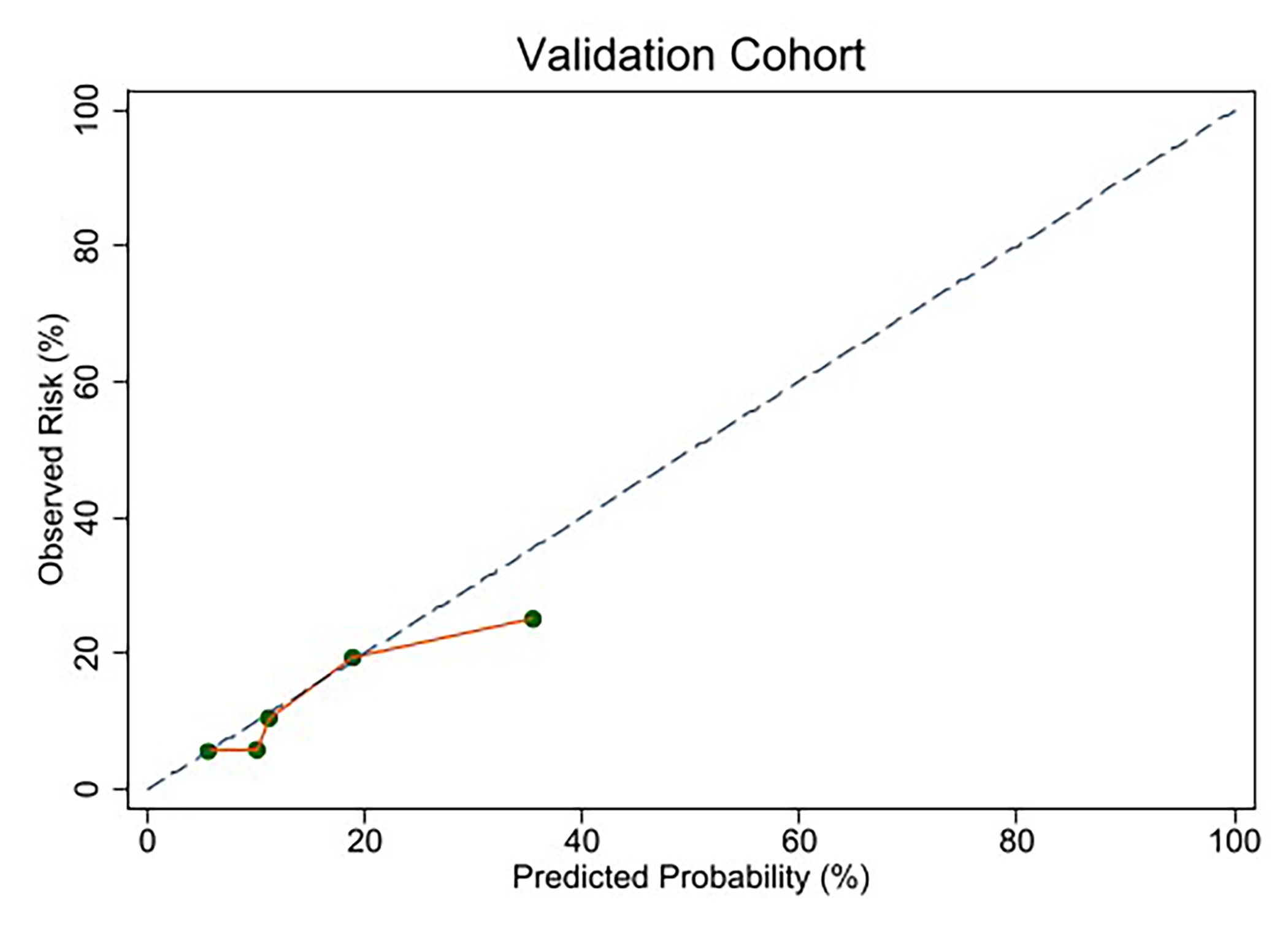

Supplement: Figures_TIF_R1.zip [file IRNF_A_2509785_SM5944.zip › Figures_TIF_R1/Figure2D.tif]

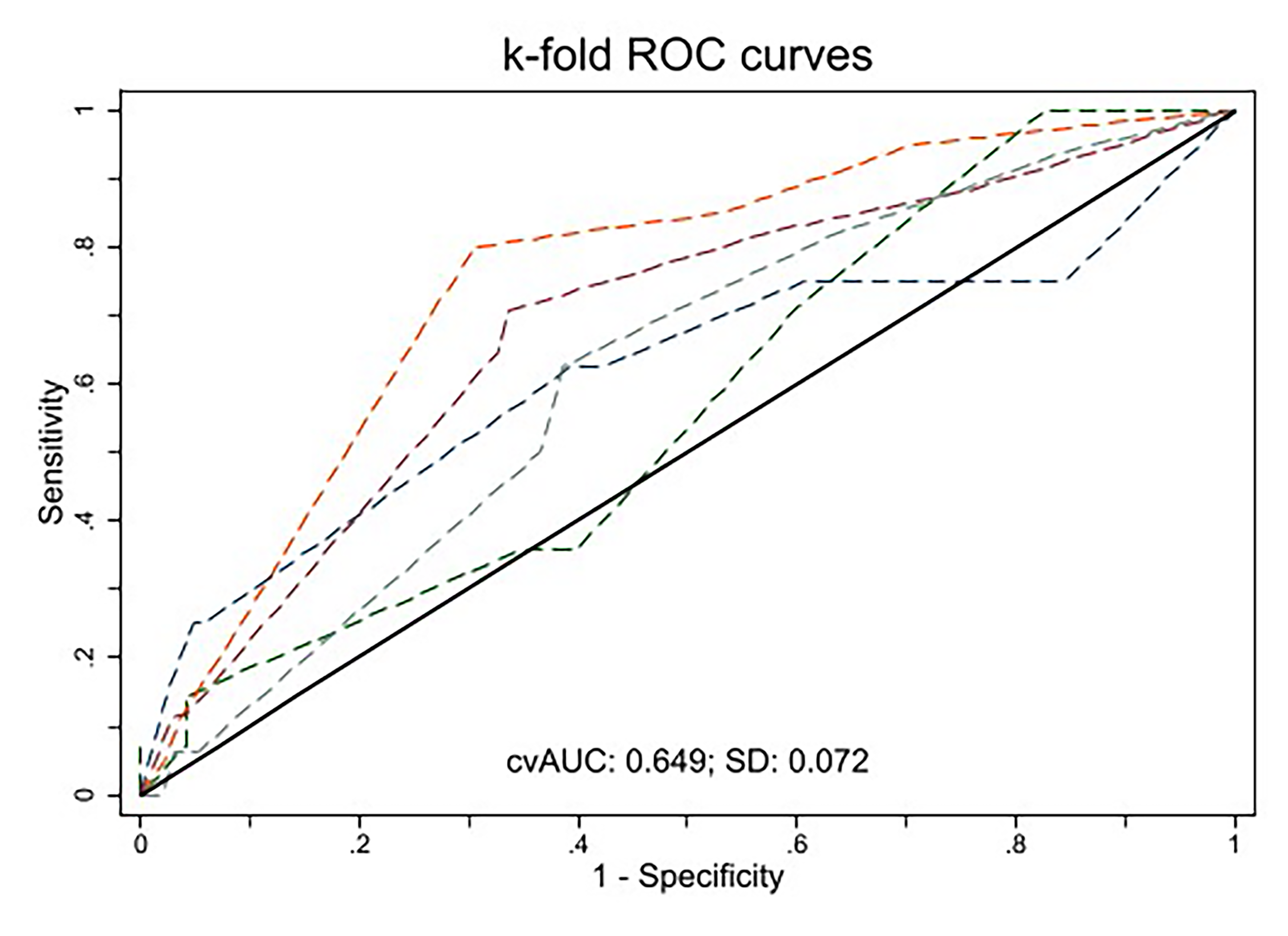

Supplement: Figures_TIF_R1.zip [file IRNF_A_2509785_SM5944.zip › Figures_TIF_R1/Figure3.tif]

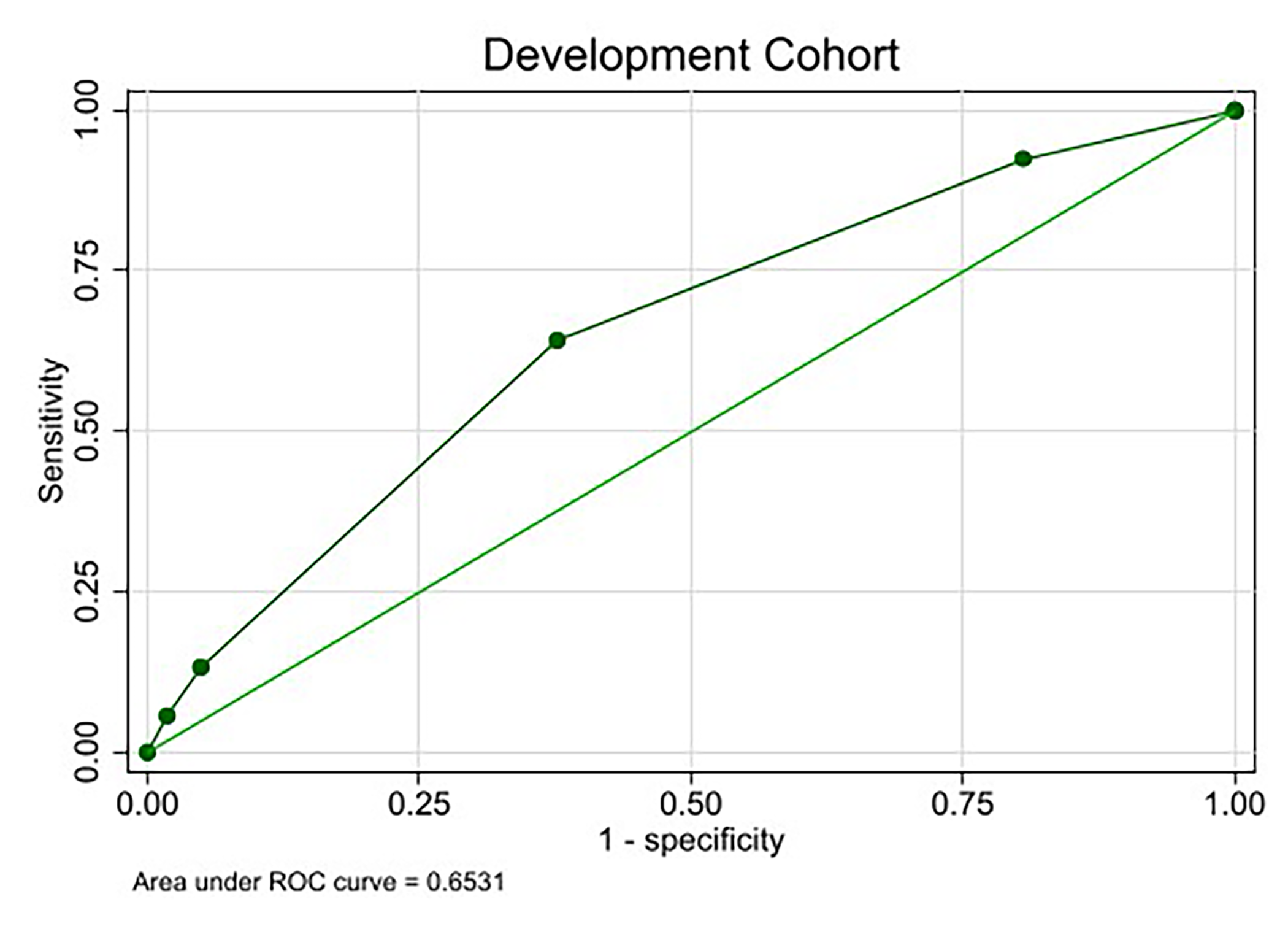

Supplement: Figures_TIF_R1.zip [file IRNF_A_2509785_SM5944.zip › Figures_TIF_R1/Figure4A.tif]

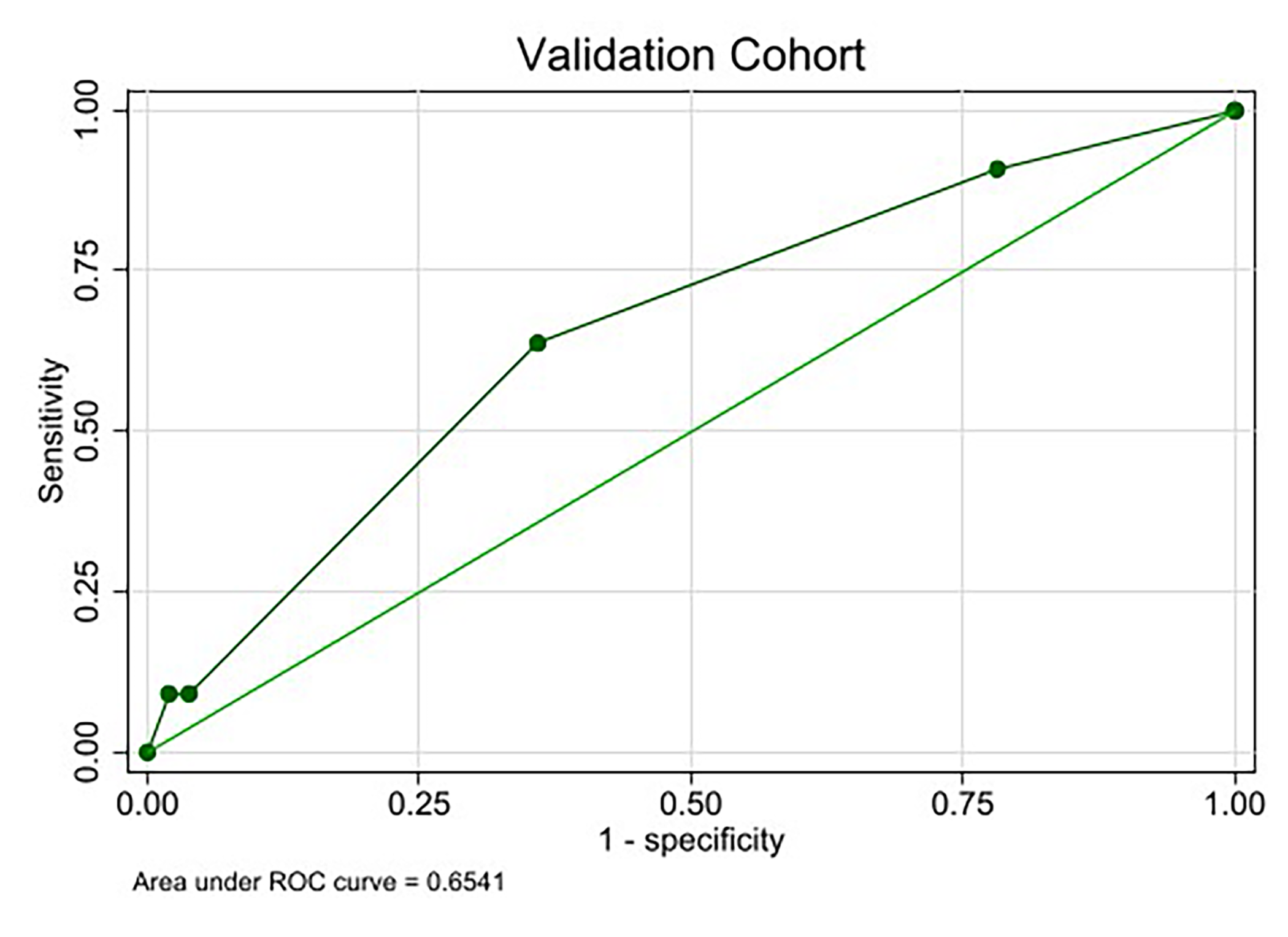

Supplement: Figures_TIF_R1.zip [file IRNF_A_2509785_SM5944.zip › Figures_TIF_R1/Figure4B.tif]

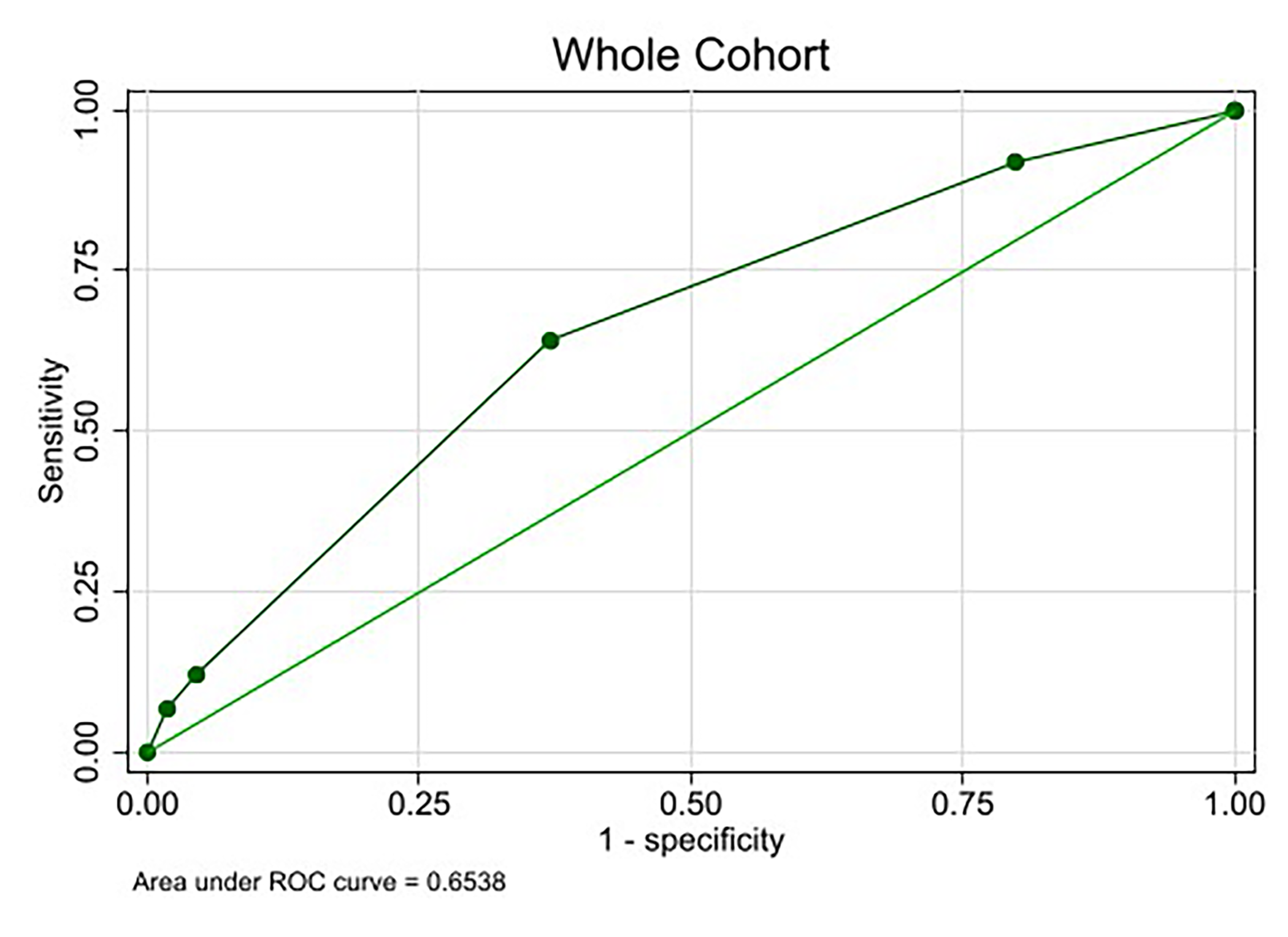

Supplement: Figures_TIF_R1.zip [file IRNF_A_2509785_SM5944.zip › Figures_TIF_R1/Figure4C.tif]

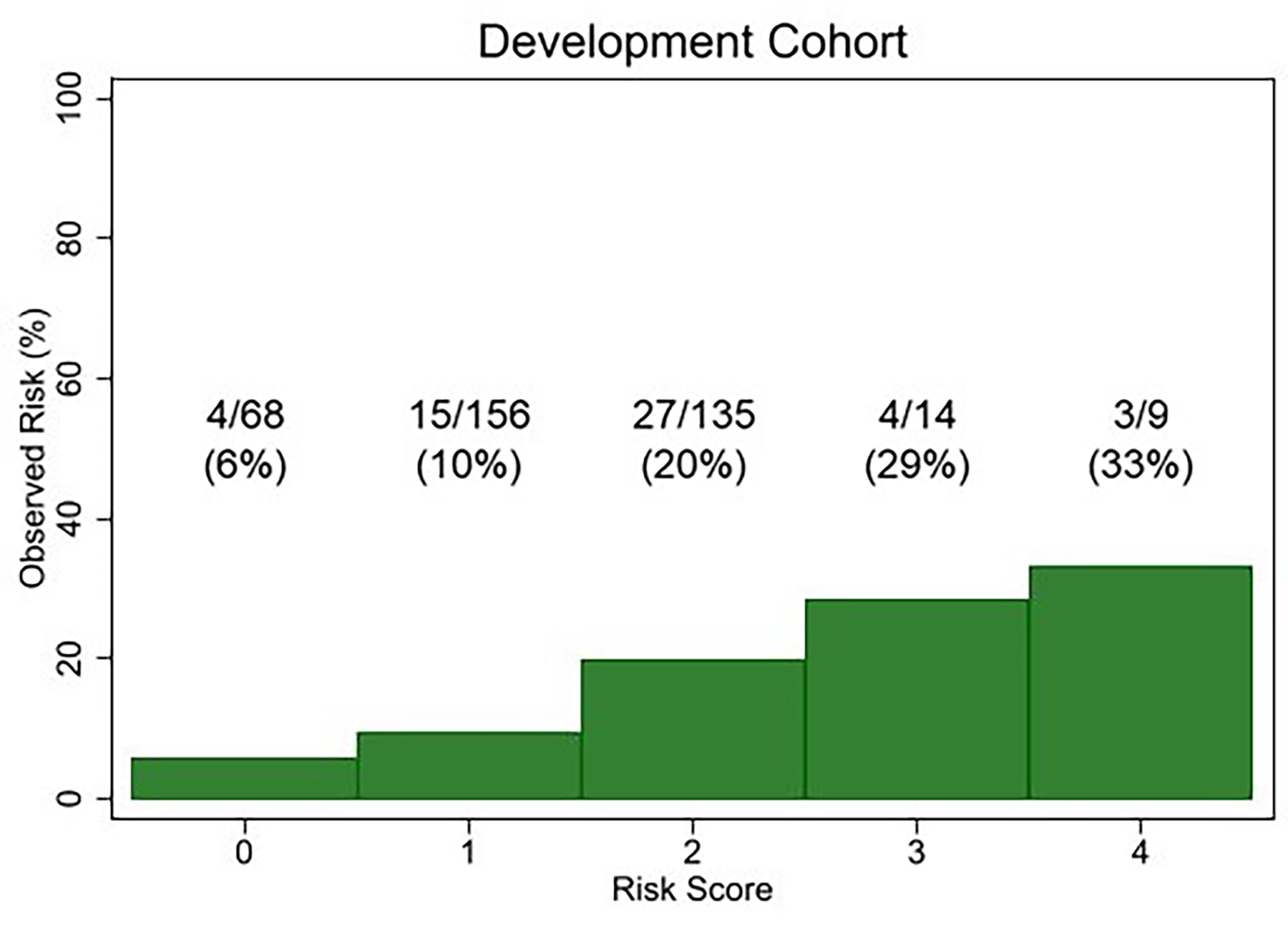

Supplement: Figures_TIF_R1.zip [file IRNF_A_2509785_SM5944.zip › Figures_TIF_R1/Figure4D.tif]

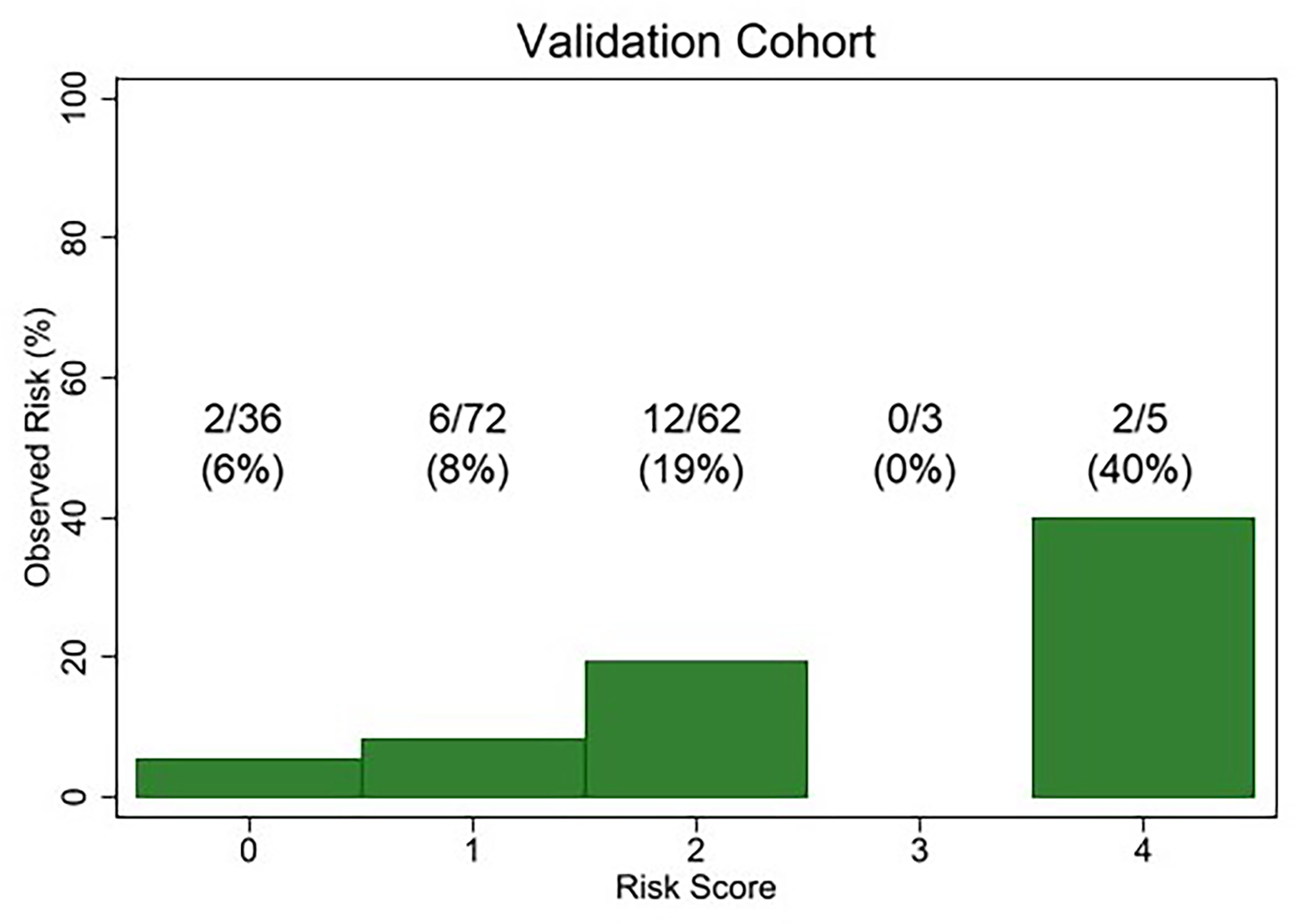

Supplement: Figures_TIF_R1.zip [file IRNF_A_2509785_SM5944.zip › Figures_TIF_R1/Figure4E.tif]

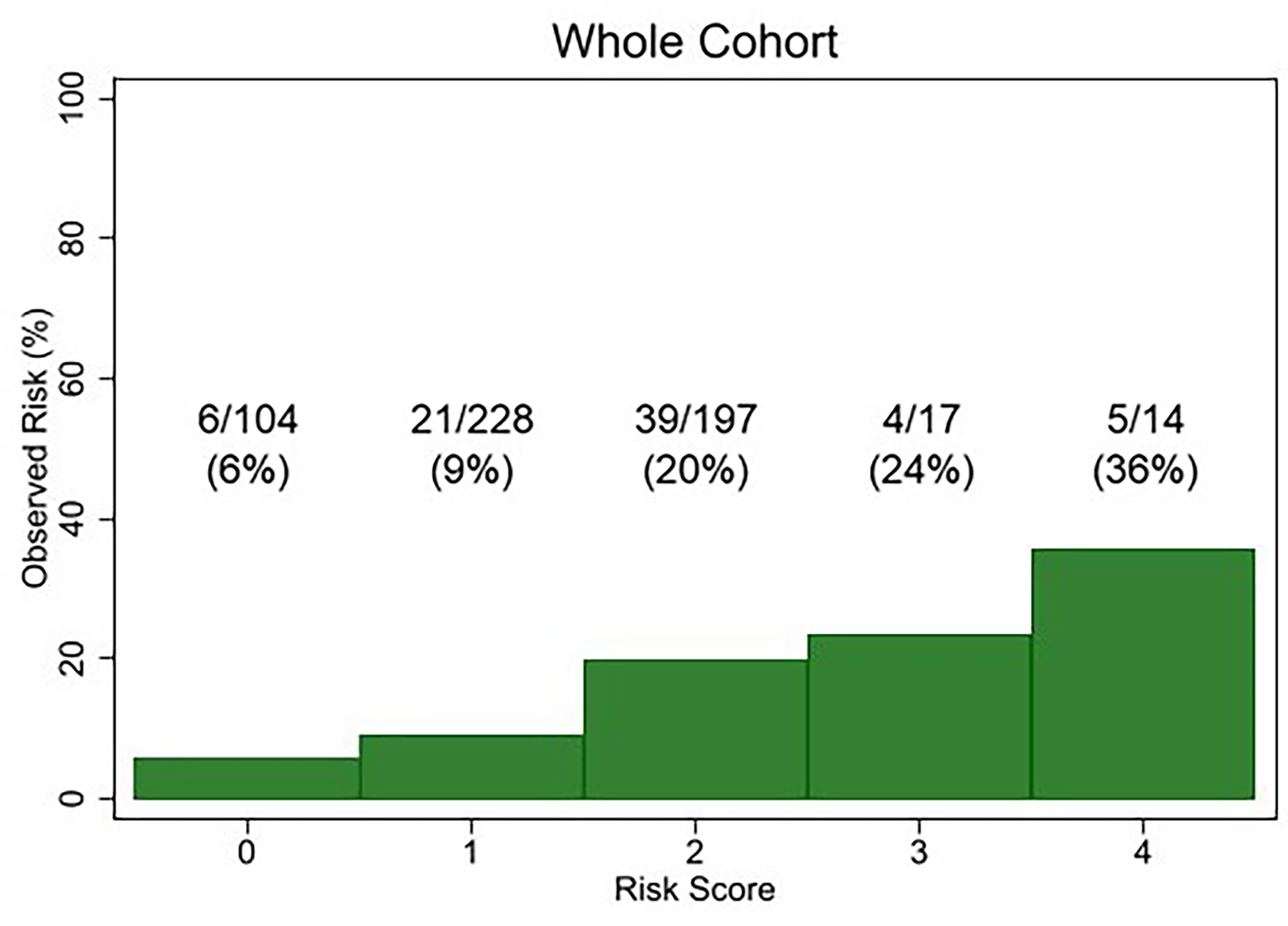

Supplement: Figures_TIF_R1.zip [file IRNF_A_2509785_SM5944.zip › Figures_TIF_R1/Figure4F.tif]

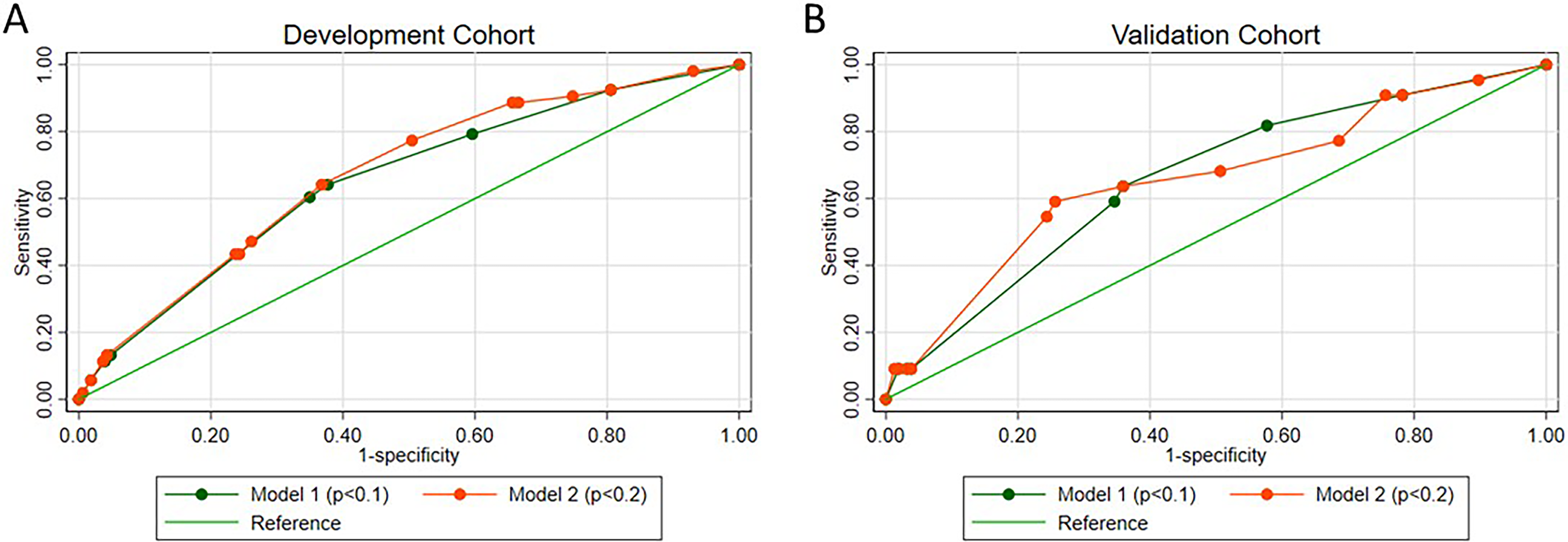

Supplement: Figures_TIF_R1.zip [file IRNF_A_2509785_SM5944.zip › Figures_TIF_R1/FigureS1.tif]

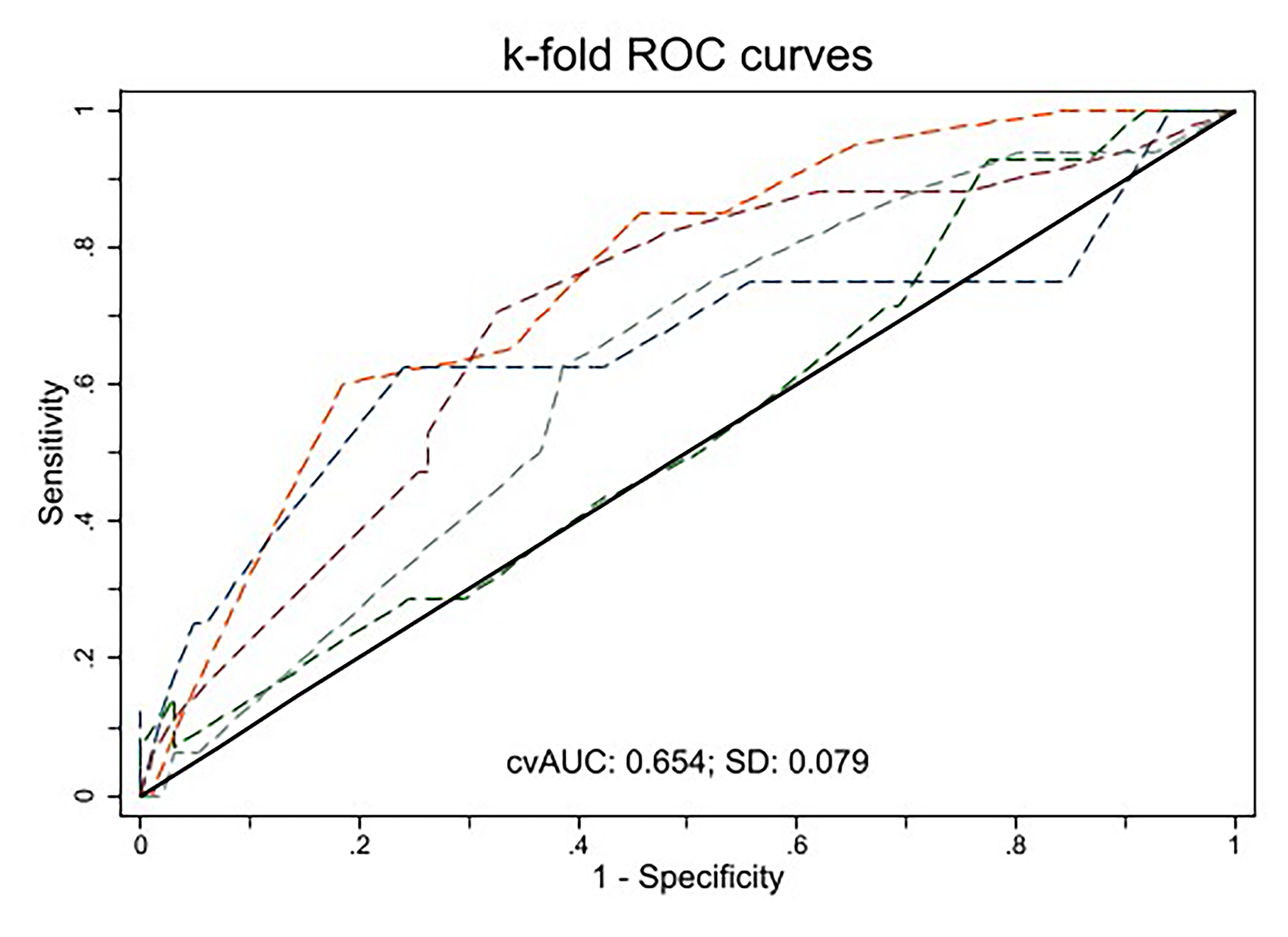

Supplement: Figures_TIF_R1.zip [file IRNF_A_2509785_SM5944.zip › Figures_TIF_R1/FigureS2.tif]

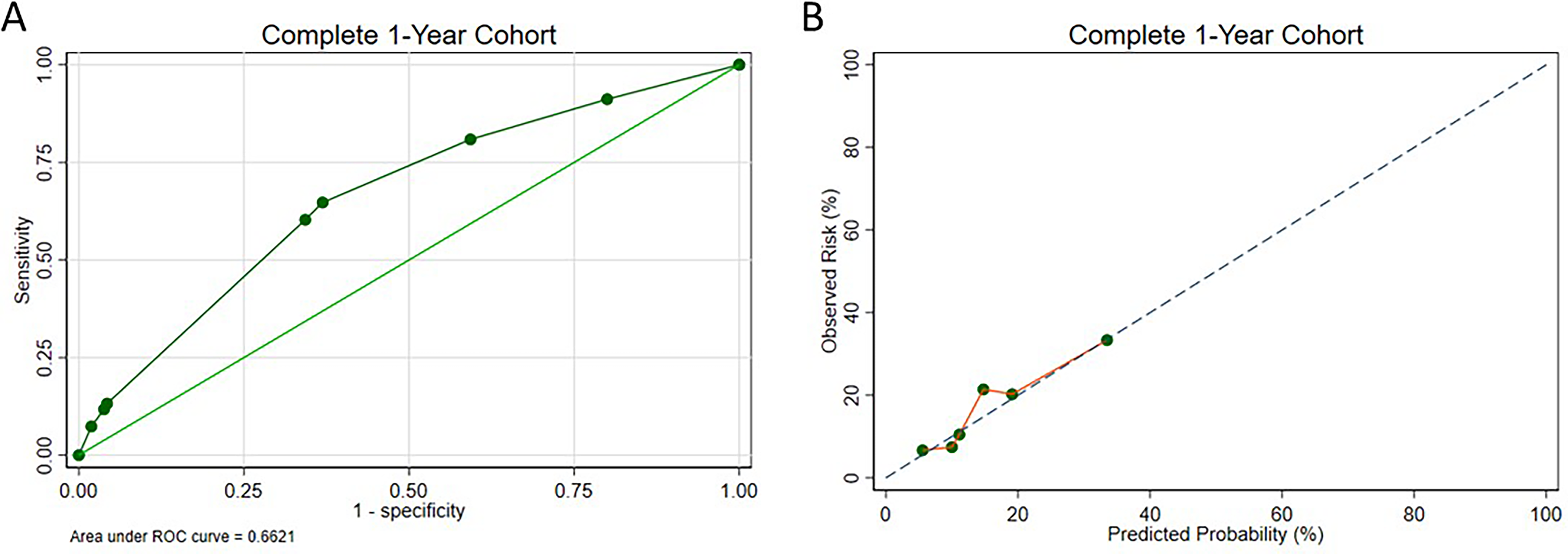

Supplement: Figures_TIF_R1.zip [file IRNF_A_2509785_SM5944.zip › Figures_TIF_R1/FigureS3.tif]

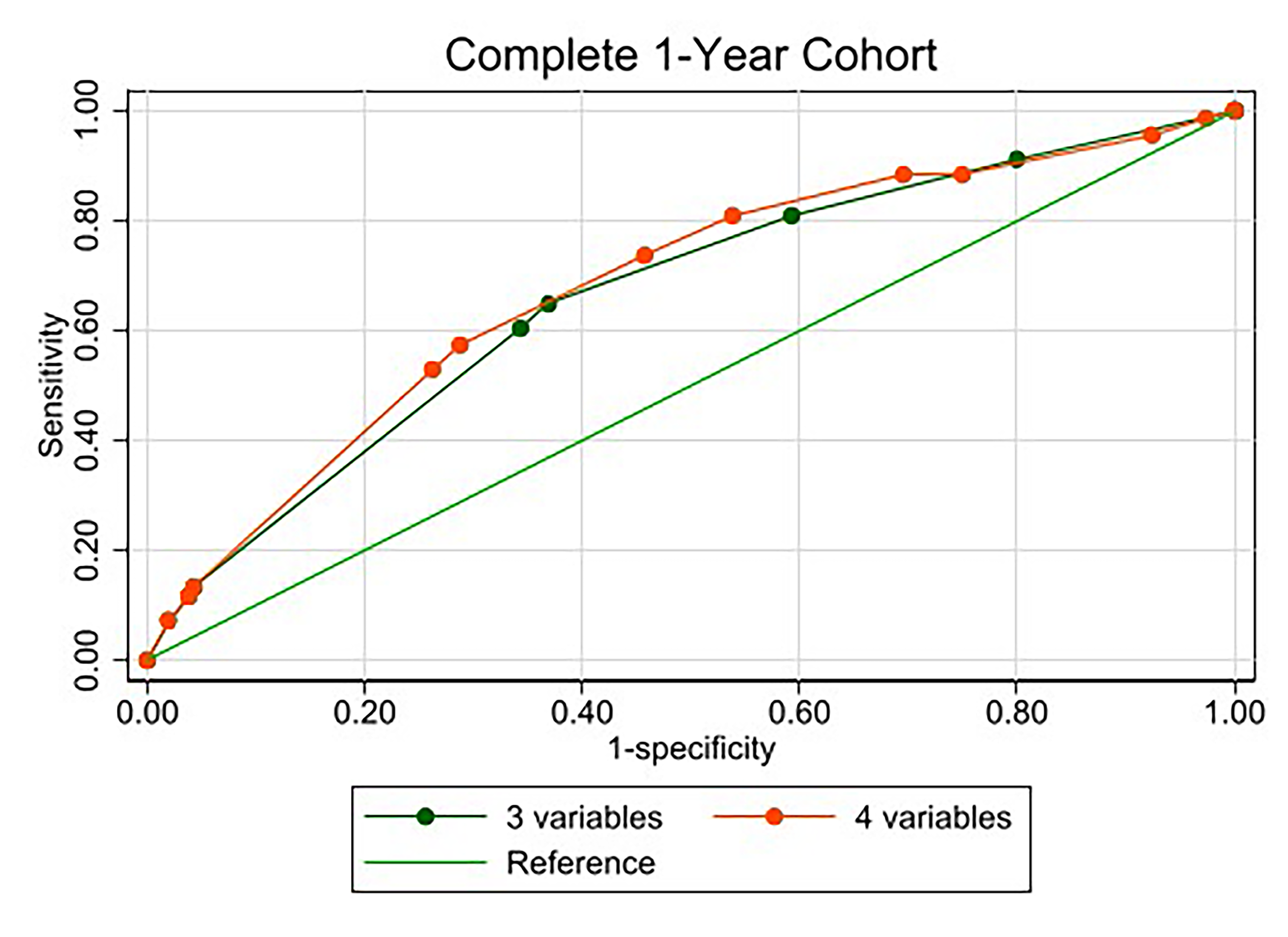

Supplement: Figures_TIF_R1.zip [file IRNF_A_2509785_SM5944.zip › Figures_TIF_R1/FigureS4.tif]

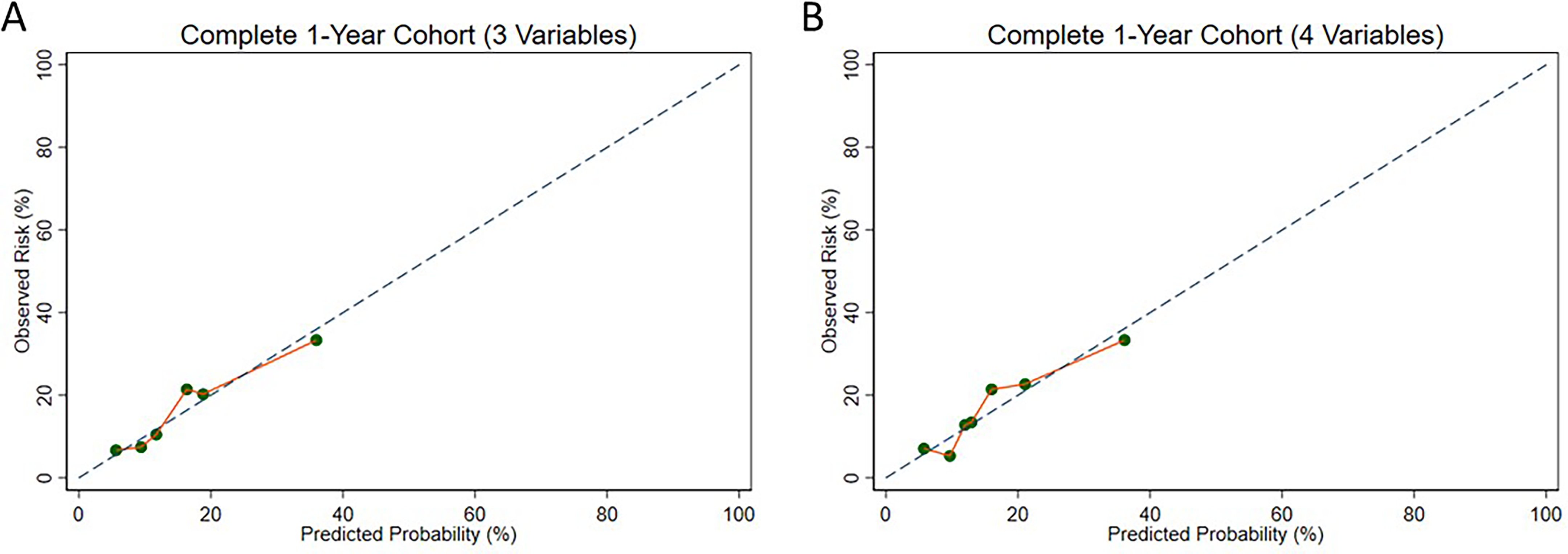

Supplement: Figures_TIF_R1.zip [file IRNF_A_2509785_SM5944.zip › Figures_TIF_R1/FigureS5.tif]

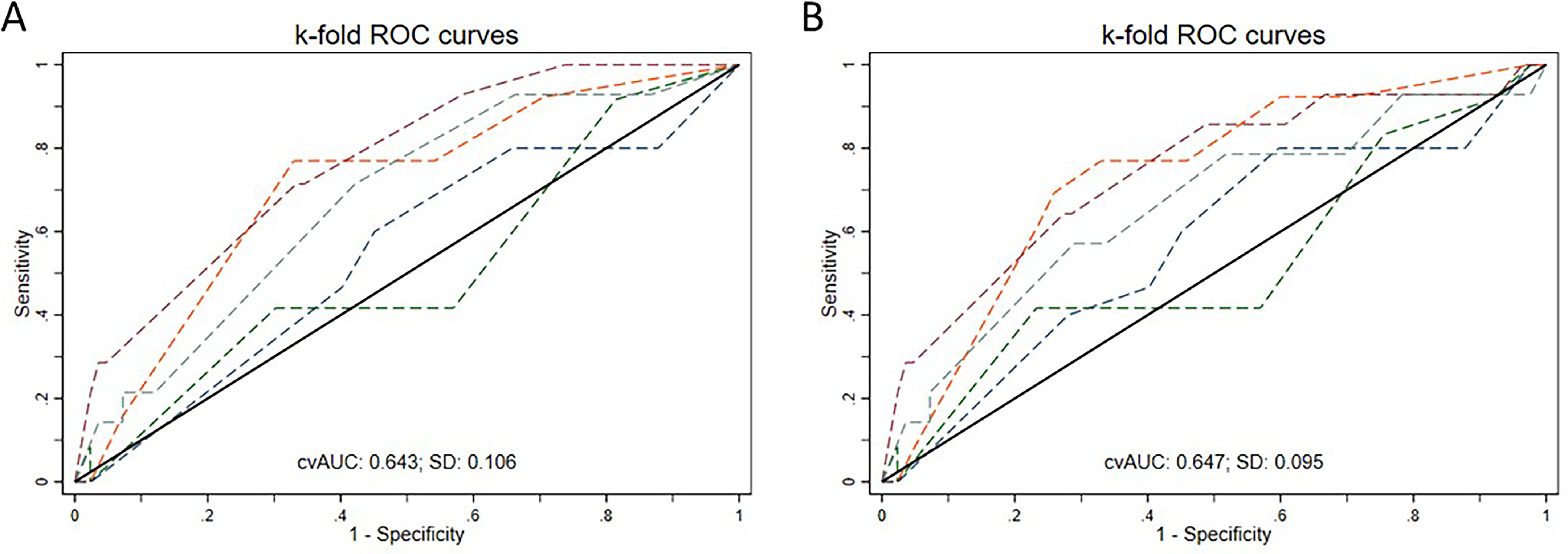

Supplement: Figures_TIF_R1.zip [file IRNF_A_2509785_SM5944.zip › Figures_TIF_R1/FigureS6.tif]
