# Supplementary material for: Development of BK polyomavirus-associated nephropathy risk prediction in kidney transplant recipients
Source: Ren Fail. 2025 May 29;47(1):2509785. doi: 10.1080/0886022X.2025.2509785 (PMC12123896; doi:10.1080/0886022X.2025.2509785)
Supplement: BKVAN_Risk_Supplement_Tables_R1.docx [file IRNF_A_2509785_SM5943.docx]

**Development of BK Polyomavirus-Associated Nephropathy Risk Prediction in Kidney Transplant Recipients**

**Supplementary Tables**

Table S1. Patient characteristics of the development cohort

Table S2. Patient characteristics of the validation cohort

Table S3. Logistic regression models created using backward selection cutoff of p<0.1 and p<0.2

Table S4. Logistic regression analysis for BK polyomavirus-associated nephropathy development on the complete 1-year follow-up cohort

**Table S1. Patient characteristics of the development cohort**

| Characteristic | Total | BKPyVAN Negative | BKPyVAN Positive | p-value |
| --- | --- | --- | --- | --- |
|  | N=382 | N=329 | N=53 |  |
| Recipient |  |  |  |  |
| Recipient age (years) | 51 (15) | 50 (15) | 53 (15) | 0.23 |
| Age (4 categories) |  |  |  | 0.25 |
| <=40 years | 113 (30%) | 102 (31%) | 11 (21%) |  |
| 40< - 50 years | 56 (15%) | 50 (15%) | 6 (11%) |  |
| 50< - 60 years | 86 (23%) | 70 (21%) | 16 (30%) |  |
| >60 years | 127 (33%) | 107 (33%) | 20 (38%) |  |
| Age (2 categories) |  |  |  | 0.055 |
| <=50 years | 169 (44%) | 152 (46%) | 17 (32%) |  |
| >50 years | 213 (56%) | 177 (54%) | 36 (68%) |  |
| Recipient sex |  |  |  | 0.016 |
| Female | 159 (42%) | 145 (44%) | 14 (26%) |  |
| Male | 223 (58%) | 184 (56%) | 39 (74%) |  |
| Race |  |  |  | 0.81 |
| White | 248 (65%) | 214 (65%) | 34 (64%) |  |
| Black | 7 (2%) | 5 (2%) | 2 (4%) |  |
| Hispanic | 53 (14%) | 46 (14%) | 7 (13%) |  |
| Asian | 13 (3%) | 11 (3%) | 2 (4%) |  |
| American Indian/Alaska Native | 15 (4%) | 12 (4%) | 3 (6%) |  |
| Native Hawaiian/Pacific Islander | 13 (3%) | 11 (3%) | 2 (4%) |  |
| Other | 12 (3%) | 12 (4%) | 0 (0%) |  |
| Unknown | 21 (5%) | 18 (5%) | 3 (6%) |  |
| Race category |  |  |  | 0.90 |
| White | 248 (65%) | 214 (65%) | 34 (64%) |  |
| Other | 134 (35%) | 115 (35%) | 19 (36%) |  |
| Body mass index (kg/m2) | 28.6 (5.4) | 28.5 (5.5) | 28.8 (5.2) | 0.72 |
| Body mass index (4 categories) |  |  |  | 0.69 |
| <25 kg/m2 | 104 (27%) | 93 (28%) | 11 (21%) |  |
| 25 - <30 kg/m2 | 127 (33%) | 107 (33%) | 20 (38%) |  |
| 30 - <35 kg/m2 | 92 (24%) | 78 (24%) | 14 (26%) |  |
| >=35 kg/m2 | 59 (15%) | 51 (16%) | 8 (15%) |  |
| Body mass index (2 categories) |  |  |  | 0.75 |
| <30 kg/m2 | 231 (60%) | 200 (61%) | 31 (58%) |  |
| >=30 kg/m2 | 151 (40%) | 129 (39%) | 22 (42%) |  |
| Dialysis duration (years) | 3 (1-5) | 3 (1-5) | 3 (1-5) | 0.38 |
| Dialysis duration category (3 categories) |  |  |  | 0.30 |
| Preemptive | 72 (19%) | 63 (19%) | 9 (17%) |  |
| <3 years | 173 (45%) | 153 (47%) | 20 (38%) |  |
| >=3 years | 137 (36%) | 113 (34%) | 24 (45%) |  |
| Cause of kidney failure |  |  |  | 0.22 |
| Diabetes | 102 (27%) | 87 (26%) | 15 (28%) |  |
| Hypertension | 54 (14%) | 42 (13%) | 12 (23%) |  |
| Glomerulonephritis | 84 (22%) | 73 (22%) | 11 (21%) |  |
| Cystic disease | 26 (7%) | 25 (8%) | 1 (2%) |  |
| Others | 116 (30%) | 102 (31%) | 14 (26%) |  |
| Cause of kidney failure category |  |  |  | 0.78 |
| Other | 280 (73%) | 242 (74%) | 38 (72%) |  |
| Diabetes | 102 (27%) | 87 (26%) | 15 (28%) |  |
| HLA mismatch | 4 (3-5) | 4 (3-5) | 4 (3-5) | 0.76 |
| HLA mismatch |  |  |  | 0.17 |
| 0 | 17 (4%) | 14 (4%) | 3 (6%) |  |
| 1 | 10 (3%) | 7 (2%) | 3 (6%) |  |
| 2 | 21 (5%) | 19 (6%) | 2 (4%) |  |
| 3 | 72 (19%) | 64 (19%) | 8 (15%) |  |
| 4 | 89 (23%) | 78 (24%) | 11 (21%) |  |
| 5 | 120 (31%) | 97 (29%) | 23 (43%) |  |
| 6 | 53 (14%) | 50 (15%) | 3 (6%) |  |
| HLA mismatch category |  |  |  | 0.84 |
| 0-3 mismatch | 120 (31%) | 104 (32%) | 16 (30%) |  |
| 4-6 mismatch | 262 (69%) | 225 (68%) | 37 (70%) |  |
| Prior kidney transplant | 34 (9%) | 25 (8%) | 9 (17%) | 0.026 |
| BKPyVAN history in prior kidney transplant | 2 (6%) | 1 (4%) | 1 (11%) | 0.44 |
| Induction antibody |  |  |  | 0.66 |
| Basiliximab | 35 (9%) | 31 (9%) | 4 (8%) |  |
| Anti-thymocyte globulin/alemtuzumab | 347 (91%) | 298 (91%) | 49 (92%) |  |
| Belatacept/Tacrolimus |  |  |  | 0.40 |
| Belatacept | 74 (19%) | 66 (20%) | 8 (15%) |  |
| Tacrolimus | 308 (81%) | 263 (80%) | 45 (85%) |  |
| Ureteral stent placement | 305 (80%) | 267 (81%) | 38 (72%) | 0.11 |
| Delayed graft function | 21 (5%) | 20 (6%) | 1 (2%) | 0.21 |
| Donor |  |  |  |  |
| Donor type |  |  |  | 0.26 |
| Living | 127 (33%) | 113 (34%) | 14 (26%) |  |
| Deceased | 255 (67%) | 216 (66%) | 39 (74%) |  |
| Cold ischemia time (hours) | 12 (9) | 12 (9) | 13 (9) | 0.64 |
| Cold ischemia time (4 categories) |  |  |  | 0.91 |
| <12 hours | 189 (49%) | 162 (49%) | 27 (51%) |  |
| 12 - <18 hours | 81 (21%) | 71 (22%) | 10 (19%) |  |
| 18 - <24 hours | 70 (18%) | 61 (19%) | 9 (17%) |  |
| >=24 hours | 42 (11%) | 35 (11%) | 7 (13%) |  |
| Cold ischemia time (2 categories) |  |  |  | 0.58 |
| <24 hours | 340 (89%) | 294 (89%) | 46 (87%) |  |
| >=24 hours | 42 (11%) | 35 (11%) | 7 (13%) |  |
| BK polyomavirus |  |  |  |  |
| Highest plasma BKPyV DNA within 1 year (copies/mL) | 0 (0-487) | 0 (0-0) | 168668 (50200-613000) | <0.001 |
| Plasma BKPyV DNA category (highest within 1 year) |  |  |  | <0.001 |
| Not detected or unquantifiable | 260 (68%) | 260 (79%) | 0 (0%) |  |
| <=1,000 copies/mL | 35 (9%) | 35 (11%) | 0 (0%) |  |
| 1,000< - 10,000 copies/mL | 34 (9%) | 34 (10%) | 0 (0%) |  |
| >10,000 copies/mL | 53 (14%) | 0 (0%) | 53 (100%) |  |
| Days from transplant to first plasma BKPyV DNA >10,000 copies/mL | 114 (81-203) | - | 114 (81-203) | - |
| Kidney biopsy performed within 1 year | 143 (37%) | 115 (35%) | 28 (53%) | 0.013 |
| Kidney biopsy performed during BKPyV DNA >10,000 copies/mL | 22 (42%) | - | 22 (42%) | - |
| Biopsy-proven BKPyVAN | 17 (4%) | 0 (0%) | 17 (32%) | - |

Values are expressed as mean (standard deviation), median (interquartile range), or number (%). Continuous variables were compared via t-tests or Mann-Whitney U tests. Categorical variables were compared via Chi-square tests.

BKPyV, BK polyomavirus; BKPyVAN, BK polyomavirus-associated nephropathy.

**Table S2. Patient characteristics of the validation cohort**

| Characteristic | Total | BKPyVAN Negative | BKPyVAN Positive | p-value |
| --- | --- | --- | --- | --- |
|  | N=178 | N=156 | N=22 |  |
| Recipient |  |  |  |  |
| Recipient age (years) | 51 (14) | 50 (14) | 55 (16) | 0.12 |
| Age (4 categories) |  |  |  | 0.26 |
| <=40 years | 53 (30%) | 48 (31%) | 5 (23%) |  |
| 40< - 50 years | 26 (15%) | 24 (15%) | 2 (9%) |  |
| 50< - 60 years | 43 (24%) | 39 (25%) | 4 (18%) |  |
| >60 years | 56 (31%) | 45 (29%) | 11 (50%) |  |
| Age (2 categories) |  |  |  | 0.21 |
| <=50 years | 79 (44%) | 72 (46%) | 7 (32%) |  |
| >50 years | 99 (56%) | 84 (54%) | 15 (68%) |  |
| Recipient sex |  |  |  | 0.055 |
| Female | 74 (42%) | 69 (44%) | 5 (23%) |  |
| Male | 104 (58%) | 87 (56%) | 17 (77%) |  |
| Race |  |  |  | 0.45 |
| White | 115 (65%) | 100 (64%) | 15 (68%) |  |
| Black | 2 (1%) | 1 (1%) | 1 (5%) |  |
| Hispanic | 27 (15%) | 25 (16%) | 2 (9%) |  |
| Asian | 10 (6%) | 8 (5%) | 2 (9%) |  |
| American Indian/Alaska Native | 4 (2%) | 3 (2%) | 1 (5%) |  |
| Native Hawaiian/Pacific Islander | 9 (5%) | 9 (6%) | 0 (0%) |  |
| Other | 5 (3%) | 4 (3%) | 1 (5%) |  |
| Unknown | 6 (3%) | 6 (4%) | 0 (0%) |  |
| Race category |  |  |  | 0.71 |
| White | 115 (65%) | 100 (64%) | 15 (68%) |  |
| Other | 63 (35%) | 56 (36%) | 7 (32%) |  |
| Body mass index (kg/m2) | 28.3 (5.1) | 28.4 (5.1) | 27.5 (4.9) | 0.43 |
| Body mass index (4 categories) |  |  |  | 0.73 |
| <25 kg/m2 | 48 (27%) | 40 (26%) | 8 (36%) |  |
| 25 - <30 kg/m2 | 67 (38%) | 59 (38%) | 8 (36%) |  |
| 30 - <35 kg/m2 | 41 (23%) | 37 (24%) | 4 (18%) |  |
| >=35 kg/m2 | 22 (12%) | 20 (13%) | 2 (9%) |  |
| Body mass index (2 categories) |  |  |  | 0.39 |
| <30 kg/m2 | 115 (65%) | 99 (63%) | 16 (73%) |  |
| >=30 kg/m2 | 63 (35%) | 57 (37%) | 6 (27%) |  |
| Dialysis duration (years) | 3 (1-5) | 3 (1-5) | 4 (2-5) | 0.33 |
| Dialysis duration category (3 categories) |  |  |  | 0.74 |
| Preemptive | 42 (24%) | 38 (24%) | 4 (18%) |  |
| <3 years | 67 (38%) | 59 (38%) | 8 (36%) |  |
| >=3 years | 69 (39%) | 59 (38%) | 10 (45%) |  |
| Cause of kidney failure |  |  |  | 0.79 |
| Diabetes | 57 (32%) | 52 (33%) | 5 (23%) |  |
| Hypertension | 26 (15%) | 22 (14%) | 4 (18%) |  |
| Glomerulonephritis | 35 (20%) | 29 (19%) | 6 (27%) |  |
| Cystic disease | 28 (16%) | 25 (16%) | 3 (14%) |  |
| Others | 32 (18%) | 28 (18%) | 4 (18%) |  |
| Cause of kidney failure category |  |  |  | 0.32 |
| Other | 121 (68%) | 104 (67%) | 17 (77%) |  |
| Diabetes | 57 (32%) | 52 (33%) | 5 (23%) |  |
| HLA mismatch | 5 (3-5) | 5 (3-5) | 4 (4-6) | 0.35 |
| HLA mismatch |  |  |  | 0.026 |
| 0 | 7 (4%) | 6 (4%) | 1 (5%) |  |
| 1 | 3 (2%) | 3 (2%) | 0 (0%) |  |
| 2 | 9 (5%) | 8 (5%) | 1 (5%) |  |
| 3 | 32 (18%) | 31 (20%) | 1 (5%) |  |
| 4 | 37 (21%) | 28 (18%) | 9 (41%) |  |
| 5 | 67 (38%) | 63 (40%) | 4 (18%) |  |
| 6 | 23 (13%) | 17 (11%) | 6 (27%) |  |
| HLA mismatch category |  |  |  | 0.096 |
| 0-3 mismatch | 51 (29%) | 48 (31%) | 3 (14%) |  |
| 4-6 mismatch | 127 (71%) | 108 (69%) | 19 (86%) |  |
| Prior kidney transplant | 11 (6%) | 8 (5%) | 3 (14%) | 0.12 |
| BKPyVAN history in prior kidney transplant | 2 (18%) | 1 (13%) | 1 (33%) | 0.42 |
| Induction antibody |  |  |  | 0.80 |
| Basiliximab | 19 (11%) | 17 (11%) | 2 (9%) |  |
| Anti-thymocyte globulin/alemtuzumab | 159 (89%) | 139 (89%) | 20 (91%) |  |
| Belatacept/Tacrolimus |  |  |  | 0.41 |
| Belatacept | 36 (20%) | 33 (21%) | 3 (14%) |  |
| Tacrolimus | 142 (80%) | 123 (79%) | 19 (86%) |  |
| Ureteral stent placement | 147 (83%) | 131 (84%) | 16 (73%) | 0.19 |
| Delayed graft function | 10 (6%) | 9 (6%) | 1 (5%) | 0.82 |
| Donor |  |  |  |  |
| Donor type |  |  |  | 0.44 |
| Living | 53 (30%) | 48 (31%) | 5 (23%) |  |
| Deceased | 125 (70%) | 108 (69%) | 17 (77%) |  |
| Cold ischemia time (hours) | 12 (9) | 12 (9) | 12 (8) | 0.88 |
| Cold ischemia time (4 categories) |  |  |  | 0.24 |
| <12 hours | 91 (51%) | 80 (51%) | 11 (50%) |  |
| 12 - <18 hours | 42 (24%) | 35 (22%) | 7 (32%) |  |
| 18 - <24 hours | 31 (17%) | 30 (19%) | 1 (5%) |  |
| >=24 hours | 14 (8%) | 11 (7%) | 3 (14%) |  |
| Cold ischemia time (2 categories) |  |  |  | 0.28 |
| <24 hours | 164 (92%) | 145 (93%) | 19 (86%) |  |
| >=24 hours | 14 (8%) | 11 (7%) | 3 (14%) |  |
| BK polyomavirus |  |  |  |  |
| Highest plasma BKPyV DNA within 1 year (copies/mL) | 0 (0-394) | 0 (0-0) | 242540 (123000-2730000) | <0.001 |
| Plasma BKPyV DNA category (highest within 1 year) |  |  |  | <0.001 |
| Not detected or unquantifiable | 128 (72%) | 128 (82%) | 0 (0%) |  |
| <=1,000 copies/mL | 11 (6%) | 11 (7%) | 0 (0%) |  |
| 1,000< - 10,000 copies/mL | 17 (10%) | 17 (11%) | 0 (0%) |  |
| >10,000 copies/mL | 22 (12%) | 0 (0%) | 22 (100%) |  |
| Days from transplant to first plasma BKPyV DNA >10,000 copies/mL | 94 (72-125) | - | 94 (72-125) | - |
| Kidney biopsy performed within 1 year | 68 (38%) | 55 (35%) | 13 (59%) | 0.031 |
| Kidney biopsy performed during BKPyV DNA >10,000 copies/mL | 11 (50%) | - | 11 (50%) | - |
| Biopsy-proven BKPyVAN | 10 (6%) | 0 (0%) | 10 (45%) | - |

Values are expressed as mean (standard deviation), median (interquartile range), or number (%). Continuous variables were compared via t-tests or Mann-Whitney U tests. Categorical variables were compared via Chi-square tests.

BKPyV, BK polyomavirus; BKPyVAN, BK polyomavirus-associated nephropathy.

**Table S3. Logistic regression models created using backward selection cutoff of p<0.1 and p<0.2**

| Covariates | Model 1 (p<0.1) | | | Model 2 (p<0.2) | | |
| --- | --- | --- | --- | --- | --- | --- |
|  | Odds ratio | 95% CI | p-value | Odds ratio | 95% CI | p-value |
| Age >50 years (vs. <=50 years) | 1.89 | (1.00, 3.57) | 0.050 | 1.93 | (1.02, 3.67) | 0.044 |
| Male (vs. female) | 2.13 | (1.10, 4.10) | 0.025 | 2.20 | (1.14, 4.26) | 0.019 |
| Prior kidney transplant, yes (vs. no) | 2.94 | (1.25, 6.95) | 0.014 | 3.01 | (1.27, 7.12) | 0.012 |
| Deceased donor (vs. living donor) | - | - | - | 1.60 | (0.82, 3.12) | 0.172 |

Logistic regression models were constructed by selecting variables via backward selection with p-values of 0.1 and 0.2.

**Table S4. Logistic regression analysis for BK polyomavirus-associated nephropathy development on the complete 1-year follow-up cohort**

| Covariates | Multivariate | | | 4 variables | | | 3 variables | | |
| --- | --- | --- | --- | --- | --- | --- | --- | --- | --- |
|  | Odds ratio | 95% CI | p-value | Odds ratio | 95% CI | p-value | Odds ratio | 95% CI | p-value |
| Age >50 years (vs. <=50 years) | 1.99 | (1.10, 3.59) | **0.023** | 1.80 | (1.03, 3.14) | **0.040** | 1.75 | (1.00, 3.05) | **0.050** |
| Male (vs. female) | 2.42 | (1.32, 4.45) | **0.004** | 2.26 | (1.25, 4.10) | **0.007** | 2.22 | (1.23, 4.02) | **0.008** |
| Race Other (vs. White) | 1.00 | (0.54, 1.84) | 0.989 |  |  |  |  |  |  |
| Body mass index >=30 kg/m2 (vs. <30 kg/m2) | 0.84 | (0.48, 1.47) | 0.539 |  |  |  |  |  |  |
| Kidney failure from diabetes (vs. other) | 0.83 | (0.43, 1.59) | 0.569 |  |  |  |  |  |  |
| Dialysis duration (vs. preemptive) |  |  |  |  |  |  |  |  |  |
| <3 years | 0.88 | (0.40, 1.93) | 0.752 |  |  |  |  |  |  |
| >=3 years | 1.28 | (0.55, 2.99) | 0.571 |  |  |  |  |  |  |
| HLA mismatch (vs. 0-3 mismatch) |  |  |  |  |  |  |  |  |  |
| 4-6 mismatch | 1.30 | (0.69, 2.46) | 0.411 |  |  |  |  |  |  |
| Prior kidney transplant, yes (vs. no) | 2.79 | (1.22, 6.36) | **0.015** | 2.90 | (1.35, 6.25) | **0.006** | 3.28 | (1.54, 6.99) | **0.002** |
| Deceased donor (vs. living donor) | 1.30 | (0.65, 2.62) | 0.459 |  |  |  |  |  |  |
| Cold ischemia time >=24 hours (vs. <24 hours) | 1.43 | (0.62, 3.30) | 0.398 |  |  |  |  |  |  |
| Anti-thymocyte globulin/alemtuzumab (vs. basiliximab) | 1.62 | (0.58, 4.48) | 0.355 |  |  |  |  |  |  |
| Tacrolimus (vs. belatacept) | 2.30 | (1.01, 5.22) | **0.047** | 1.96 | (0.89, 4.33) | **0.097** |  |  |  |

Multivariate logistic regression was performed on the complete 1-year follow-up cohort (n=488). Backward selection with a p-value threshold of 0.1 identified 4 variables (age, male, prior kidney transplant, and tacrolimus use). The 3-variable model, which includes the final model predictors in the primary analysis, is shown as a reference. Bold values denote p-values <0.1.
